# Understanding the impact of natural disasters on post-traumatic stress disorder and depression symptoms: An examination of counterfactual displacement scenarios

**DOI:** 10.1371/journal.pmen.0000317

**Published:** 2026-06-18

**Authors:** Mohammed Abba-Aji, Gregory Cohen, Salma Abdalla, Ruochen Wang, Jaimie Gradus, Sandro Galea

**Affiliations:** 1 Department of Epidemiology, Boston University School of Public Health, Boston, Massachusetts, United States of America; 2 Washington University School of Public Health, St. Louis, Missouri, United States of America; 3 School of Dentistry, University of Utah, Salt Lake City, Utah, United States of America; 4 School of Public Health and Health Sciences, University of Massachusetts Amherst, Amherst, Massachusetts, United States of America; Sigmund Freud University Vienna, AUSTRIA

## Abstract

Displacement following extreme weather events is a growing concern, yet evidence on its mental health impacts remains limited. In August 2017, Hurricane Harvey caused extensive flooding and damage in Houston, Texas, leading to substantial displacement. In this study, we take a counterfactual approach to examine how displacement scenarios affect post-traumatic stress disorder (PTSD) and depression symptoms among impacted residents. We conducted a cross-sectional survey of adult Houston residents who experienced Hurricane Harvey. Using Augmented Inverse Probability Weighting and Inverse Probability Weighted Regression Adjustment within a causal inference framework, we estimated the association between displacement and PTSD and depression symptom scores among residents affected by Hurricane Harvey. First, we estimated the Potential Outcome Means (POmeans) under counterfactual scenarios of displacement and non-displacement. We then computed the Average Treatment Effect (ATE) and the Average Treatment Effect on the Treated (ATT) for both PTSD and depression. Covariates included demographic factors, socioeconomic status, and self-reported physical health. POmeans indicated that displaced individuals had elevated PTSD and depression symptom levels compared with a counterfactual scenario in which they were not displaced. Displacement was associated with higher PTSD symptom scores (ATE: 5.88 points [95% CI, 3.37–8.39]; ATT: 5.43 [95% CI, 2.65–8.20]) and higher depression symptom scores (ATE: 1.93 [95% CI, 0.99–2.87]; ATT: 2.12 [95% CI, 1.04–3.20]). Using causal inference methods, our analysis suggests that displacement is associated with worsened PTSD and depression symptomatology. While our doubly robust approach strengthens inference, these estimates should be interpreted as associations given the potential for residual confounding from unmeasured variables. As climate-related disasters intensify, these results highlight the importance of proactive policies and mental health interventions that specifically address the psychological toll of displacement. Future studies using longitudinal designs could further elucidate the causal pathways linking disaster-related displacement to long-term mental health outcomes.

## Introduction

Globally, the number of extreme weather events (such as hurricanes, floods, and wildfires) has more than doubled over the past 40 years due to climate change [1]. In the United States, the trend is similar, with a significant increase in the number of disasters in which damage costs exceed $1 billion during the past three decades [2]. The intensity of these events has also increased, leading to more severe impacts on affected populations and greater challenges for disaster response and recovery efforts [3]. Consequently, natural disasters are often associated with widespread displacement. The Internal Displacement Monitoring Centre (IDMC) reports that in 2024, natural disasters displaced 75.9 million people globally [4]. The United States reported 11 million disaster-related displacements (movements) in 2024 — the most ever recorded for a single country [5]. Losing one’s home after a disaster often also means losing security, social networks, and access to essential resources [6]. These stressors significantly contribute to the onset or worsening of mental health conditions, including post-traumatic stress disorder (PTSD) and depression [7,8]. Further, studies have demonstrated a strong link between displacement due to natural disasters and an increased risk of mental health disorders, particularly depression and PTSD. For example, a few cross sectional studies have shown that displaced persons are more likely to experience mental health issues—specifically, depression, PTSD, and anxiety symptoms—compared to those who were not displaced [9,10].

While there is evidence of the relation between displacement and poor mental health, our understanding of the mental health impact of displacement on the broader population—including both those directly displaced and those who may have been affected indirectly—remains limited (10). Even when the role of displacement in shaping mental health outcomes has been studied, most studies have relied on convenience samples, such as those from disaster relief centers or emergency response lists. These samples often do not accurately represent the entirety of the population displaced by natural disasters [11,12], particularly those who do not seek formal assistance [13]. This reliance on non-representative samples may introduce selection bias, limiting our ability to generalize findings across the broader displaced populations. Even among studies that have considered more representative samples, most studies have not used methods that estimate the causal effect of displacement on such mental health indicators [10,14]. In addition, these often rely on binary diagnostic measures for mental health indicators, which may not capture subtle variations in symptom severity [10]. Therefore, there is a need to use causal inference methods to estimate the impact of displacement on mental health indicators among representative samples while also recognizing that health outcomes exist on a continuum rather than in binary terms [15].

To address these gaps in the evidence, we examined the relationship between displacement and symptoms of two mental health disorders—PTSD and depression—among U.S. communities affected by natural disasters. Recent research in Houston found that more than three years after Hurricane Harvey, the prevalence of major depression was 5.8% and PTSD was 12.6%, highlighting the long-term mental health impacts of major disasters [16]. Building on this, we analyze data from a representative sample of residents of Houston, Texas, who experienced Hurricane Harvey in 2017, conceptualizing our study within a causal inference framework to better understand the potential impact of displacement on mental health outcomes.

## Methods

### Ethics statement

This study was approved by the Boston University Medical Campus Institutional Review Board (IRB H-39516). Formal informed consent was obtained when participants agreed to participate and completed the study survey. A waiver of documentation of consent was granted because the research presented no more than minimal risk to participants and involved no procedures for which written consent would normally be required outside of the research context. We followed the STROBE reporting guideline for observational studies [17].

### Study design and participants

We conducted an observational survey (the Houston Trauma and Recovery Study) employing address-based sampling. A total of 12,009 recruitment letters were mailed, with 11,110 found eligible and 1,266 individuals responding. Of these, 1,084 participants resided in Houston during Hurricane Harvey and met study criteria. Individuals were sampled from 88 super-neighborhoods—geographically defined areas established by the City of Houston to foster collaboration and streamline service delivery [18]. Data were collected between 17/11/2020–24/08/2021. Fieldwork was administered by Ipsos.

Survey weights were constructed in four stages to ensure representativeness of the target population. First, base weights were computed as the inverse of each address’s probability of selection into the survey. Second, weights were adjusted for the probability of survey response. Third, weights accounted for neighborhood size and the number of adults per household. Fourth, weights were calibrated via iterative proportional fitting (raking) to align marginal distributions of sex, race/ethnicity, age group, and educational attainment with American Community Survey estimates for the Houston Metropolitan Statistical Area [16,19]. These survey weights were applied to all descriptive analyses (Tables 1, 2, and S1 Appendix). For treatment effect estimation, the AIPW and IPWRA estimators construct their own inverse probability weights from a propensity score model that adjusts for the same demographic and socioeconomic factors targeted by the survey weights, plus additional covariates (see *Statistical analysis* below). This approach provides doubly robust estimates that account for confounding through both the propensity score and outcome regression components.

**Table 1:** Sociodemographic characteristics by displacement status (N = 1,084).

Characteristic	Not Displaced (n = 910)	Displaced (n = 174)	Total (N = 1,084)	p-value
Gender				0.775
Male	323 (48.23%)	57 (47.36%)	380 (48.11%)	
Female	581 (50.53%)	116 (51.25%)	697 (50.63%)	
Other	6 (1.25%)	1 (1.39%)	7 (1.27%)	
Age Category				0.620
18–24	44 (12.15%)	7 (11.16%)	51 (12.02%)	
25–34	120 (23.28%)	16 (15.50%)	136 (22.21%)	
35–44	158 (13.94%)	34 (28.11%)	192 (15.88%)	
45–54	128 (15.77%)	28 (16.74%)	156 (15.90%)	
55–64	163 (19.36%)	29 (12.33%)	192 (18.40%)	
65–74	188 (9.18%)	32 (8.39%)	220 (9.07%)	
75–84	81 (4.62%)	21 (4.92%)	102 (4.66%)	
85+	28 (1.70%)	7 (2.85%)	35 (1.86%)	
Education				0.238
Less than high school	84 (16.56%)	20 (21.73%)	104 (17.27%)	
High school graduate or GED	150 (29.66%)	30 (30.72%)	180 (29.81%)	
Some college or technical training	216 (16.77%)	50 (12.60%)	266 (16.20%)	
College or graduate degree	460 (37.01%)	74 (34.94%)	534 (36.72%)	
Race/Ethnicity				0.196
White, non-Hispanic	359 (38.71%)	54 (28.43%)	413 (37.30%)	
Black, non-Hispanic	220 (12.08%)	51 (23.95%)	271 (13.71%)	
Other or 2 + Races, non-Hispanic	74 (5.74%)	15 (5.54%)	89 (5.71%)	
Hispanic	257 (43.46%)	54 (42.09%)	311 (43.27%)	
Marital Status				0.441
Divorced, separated, or widowed	244 (14.10%)	55 (17.07%)	299 (14.51%)	
Never married	257 (33.82%)	47 (37.57%)	304 (34.34%)	
Married	406 (52.08%)	72 (45.36%)	478 (51.15%)	
Physical Health				0.137
Excellent	129 (15.60%)	25 (18.53%)	154 (15.99%)	
Very good	315 (35.17%)	46 (27.21%)	361 (34.09%)	
Good	302 (33.30%)	64 (25.93%)	366 (32.30%)	
Fair	143 (14.27%)	30 (24.25%)	173 (15.62%)	
Poor	20 (1.67%)	8 (4.08%)	28 (1.99%)	
Living Place				0.673
Own it	579 (57.27%)	102 (49.42%)	681 (56.20%)	
Rent it	301 (38.64%)	63 (48.50%)	364 (40.00%)	
Living with family/owned by family	16 (2.62%)	4 (0.44%)	20 (2.32%)	
Other	11 (1.46%)	3 (1.65%)	14 (1.49%)	
Household Income in 2019				0.011
$24,999 or less	192 (27.02%)	59 (31.01%)	251 (27.56%)	
$25,000–$49,999	196 (21.91%)	30 (22.53%)	226 (22.00%)	
$50,000–$74,999	125 (12.51%)	18 (12.62%)	143 (12.53%)	
$75,000–$99,999	84 (9.40%)	19 (9.08%)	103 (9.36%)	
$100,000–$149,999	94 (10.69%)	13 (7.09%)	107 (10.20%)	
$150,000–$199,999	58 (6.10%)	6 (2.24%)	64 (5.57%)	
$200,000 or more	112 (12.37%)	21 (15.44%)	133 (12.79%)	

Data presented as unweighted n (weighted %). Percentages are weighted to be representative of the target population. P-values from Pearson chi-square tests. Missing values excluded from percentage calculations.

**Table 2:** Mental health outcomes by displacement status.

Outcome	Total Sample (N = 1,084)	Not Displaced (n = 910)	Displaced (n = 174)	p-value
PTSD Score				<0.001
Median [IQR]	7 [1, 17]	6 [2, 16]	10 [4, 27]	
Depression Score	N = 1,081			<0.001
Median [IQR]	2 [0, 7]	2 [0, 8]	4 [1, 11]	

IQR = interquartile range. PTSD (post-traumatic stress disorder) symptoms measured using the PTSD Checklist for DSM-5 (PCL-5; range 0–80). Depression symptoms measured using the Patient Health Questionnaire-9 (PHQ-9; range 0–27). P-values from Mann-Whitney U tests due to non-normal distributions.

### Inclusion/exclusion criteria and final sample

Participants had to be at least 18 years old, reside in Houston during Hurricane Harvey, and complete core survey items on displacement and mental health. After applying propensity score approaches, 1,084 participants were retained. For analysis, 1,018 participants contributed data on post-traumatic stress disorder (PTSD) scores, and 1,015 contributed data on depression scores.

### Exposure variable: Displacement status

Displacement status was determined based on self-reported data indicating whether participants were displaced due to Hurricane Harvey. Participants who reported leaving their homes because of damage following the hurricane were classified as displaced (displaced group, n = 174), while those who did not leave their homes were classified as non-displaced (non-displaced group, n = 910).

### Outcome measures: PTSD and depression symptom scores

PTSD symptoms, over the past month, were assessed using the PTSD Checklist for DSM-5 (PCL-5), a validated 20-item self-report measure with a total symptom severity score ranging from 0 to 80. Higher symptom scores indicate more severe PTSD symptoms. The PCL-5 has demonstrated strong reliability in previous studies (Cronbach’s alpha = 0.94) and is widely used in disaster and trauma research [16,20].

Depression symptoms, over the past two weeks, were measured using the Patient Health Questionnaire-9 (PHQ-9), a validated 9-item self-report instrument with a total symptom severity score ranging from 0 to 27. Higher symptom scores indicate more severe depressive symptoms. The PHQ-9 has shown good reliability (Cronbach’s alpha = 0.89) and is a commonly used tool in epidemiological studies of depression [16,21].

### Covariates

To adjust for potential confounding factors that may influence both displacement status and mental health indicators, we selected covariates based on theoretical considerations and prior literature [10,11,22]. The following covariates were included in the analysis: gender (male, female, or other), age category (18–24, 25–34, 35–44, 45–54, 55–64, 65–74, 75–84, or 85+), educational attainment (less than high school, some college or technical training, college or graduate degree), race/ethnicity (White, non-Hispanic; Black, non-Hispanic; Hispanic; other or two or more races, non-Hispanic), marital status (divorced, separated, or widowed; never married; married), household income (≤$24,999, $25,000–$49,999, $50,000–$74,999, $75,000–$99,999, $100,000–$149,999, $150,000–$199,999, or ≥$200,000), self-reported physical health (excellent, very good, good, fair, or poor), and living condition (own, rent, live with family/friends, or other).

### Statistical analysis

We assessed the association between displacement due to home damage following Hurricane Harvey and PTSD and depression symptom scores among residents of Houston, Texas. Using augmented inverse probability weighting (AIPW) and inverse probability weighted regression adjustment (IPWRA) to adjust for potential confounders—including gender, age, education, race, marital status, income, living situation, and physical health status—we analyzed data from 1,018 participants for PTSD symptoms and 1,015 participants for depression symptoms.

Both AIPW and IPWRA methods combine propensity score weighting with outcome regression modeling [23]. These doubly robust approaches provide consistent estimates even if one of the models—the propensity score model or the outcome regression model—is misspecified. In our analysis, the propensity score model was specified using logistic regression to estimate the probability of displacement given covariates, while the outcome model was specified using linear regression to estimate the relation between displacement and mental health indicators, adjusting for covariates.

Although both AIPW and IPWRA can estimate either the Average Treatment Effect (ATE) or the Average Treatment Effect on the Treated (ATT), we report AIPW for the ATE and IPWRA for the ATT as our primary specifications. To verify that this choice does not influence the conclusions, we estimated all four estimator–estimand combinations (AIPW-ATE, AIPW-ATT, IPWRA-ATE, IPWRA-ATT) and confirmed that both estimators yield substantively identical results for both estimands (Table B in S1 Appendix). We estimated standard errors for all treatment effects using bootstrap methods with 1,000 replications to ensure robust inference that accounts for sampling variability [24].

Missing data were minimal (<5% for all variables). Missing values were addressed through imputation in a preprocessing step prior to analysis; the imputation model included all variables used in the analysis.

### Estimand and measure of effect

In our analysis, we adopt the potential outcomes framework [25,26]. Each individual *i* is assumed to have two potential outcomes; Y_₀_(0), the mental health outcome (e.g., PTSD or depression symptom score) if individual *i* is not displaced; and Y_₀_(1), the mental health outcome if individual *i* is displaced. We denote the exposure indicator by D, where D = 1 if the individual was displaced and D = 0 otherwise. Using these definitions, we derive our estimands.

#### Potential outcome means (POmeans).

E[Y(0)] denotes the average outcome under the non-displacement scenario.

E[Y(1)] denotes the average outcome under the displacement scenario.

E[Y(1) | D = 1] is the observed mean outcome among displaced individuals, while E[Y(0) | D = 1] is their counterfactual mean outcome had they not been displaced.

#### Average treatment effect (ATE).


$$\begin{array}{c} \text{ATE}=\text{E}[\text{Y}(\text{1})]-\text{E}[\text{Y}(\text{0})] \end{array}$$

This represents the expected difference in outcomes if every individual were displaced compared to if no one were displaced.

#### Average treatment effect on the treated (ATT).


$$\begin{array}{c} \text{ATT}=\text{E}[\text{Y}(\text{1})\;|\text{ D}=\text{1}]-\text{E}[\text{Y}(\text{0})\;|\text{ D}=\text{1}] \end{array}$$

This quantifies the effect of displacement specifically for those who were actually displaced by comparing their observed outcome to the counterfactual outcome had they not been displaced [26,27].

These notations underpin our analytical approach, which employs both the AIPW and IPWRA methods to estimate the association between displacement and mental health outcomes within a counterfactual framework. The validity of a causal interpretation of these estimates depends on the assumption of no unmeasured confounding conditional on included covariates—an assumption we evaluate but cannot fully verify. In our analysis, AIPW combines propensity score weighting with outcome regression modeling [27], while IPWRA integrates inverse probability weighting with regression adjustment to provide robust estimates of the treatment effect.

### Robustness checks and covariate balance

To assess the effectiveness of the propensity score weighting, we evaluated covariate balance using multiple diagnostics. First, we computed standardized mean differences (SMDs) for all covariates before and after weighting, with adequate balance indicated by |SMD| < 0.10 and variance ratios between 0.80 and 1.25 (Table C in S1 Appendix). Second, we ran an overidentification test for covariate balance using the *tebalance overid* command in Stata 19.5 [28], where a non-significant p-value (p > 0.05) indicates that the weighting procedure successfully balanced the covariates. Third, we generated balance plots to visually compare the propensity score distributions in the non-displaced and displaced groups before and after weighting. Fourth, we examined the distribution of inverse probability weights and computed the effective sample size (ESS) using Kish’s formula to assess whether extreme weights were reducing inferential precision (Table D in S1 Appendix).

All statistical analyses were conducted using Stata version 19.5 [28].

## Results

Participant characteristics are shown in Table 1. To assess representativeness, we compared our sample demographics with the adult Houston population using American Community Survey data (Table A in S1 Appendix). Our weighted sample closely aligned with Houston population demographics, supporting the generalizability of our findings. The sociodemographic characteristics of the 1,084 participants by displacement status. Displaced (n = 174) and non-displaced (n = 910) respondents were similar across most sociodemographic factors, including gender, age, education, race/ethnicity, marital status, living condition, and self-reported physical health. The only statistically significant difference observed was in household income (p = 0.011), with a higher proportion of lower-income households among the displaced group. The median PTSD symptom score for the overall sample was 7 (interquartile range [IQR]: 1–17), and the median depression symptom score was 2 (IQR: 0–7) (Table 2). Due to the non-normal distribution of the PTSD and depression symptom scores, we used the Mann-Whitney U test to compare the distributions between displaced and non-displaced persons. The median PTSD symptom score among displaced persons was 10 (IQR: 4–27), whereas non-displaced persons had a median symptom score of 6 (IQR: 2–16). For depression, displaced persons had a median score of 4 (IQR: 1–11), while non-displaced persons had a median score of 2 (IQR: 0–8). The Mann-Whitney U test showed a significant difference in PTSD and depression symptom scores between the groups (p < 0.001), suggesting that displaced persons experienced higher PTSD and depressive symptoms compared to those not displaced (Table 2).

### Association between displacement and PTSD and depression symptom scores

Under the non-displacement scenario (Y(0)), the estimated mean PTSD score was 10.85 (95% CI, 9.92–11.78). In the ATE scenario (Y(1)), where displacement occurs universally, the mean PTSD score would be 16.73 (95% CI, 14.35–19.10), indicating a shift toward moderate symptom severity. Among those who were actually displaced, the observed mean PTSD score (Y(1) | D = 1) was 16.91 (95% CI, 14.84–19.00), whereas their counterfactual mean had they not been displaced (Y(0) | D = 1) was 11.49 (95% CI, 10.01–12.96).

Next, we report the effects of displacement on PTSD. The ATE for PTSD was estimated at 5.88 points (95% CI, 3.37–8.39), suggesting that if everyone were displaced, PTSD scores would increase by nearly six points compared to a scenario where no one was displaced. Focusing on those who were actually displaced, the ATT indicated an increase of 5.43 points (95% CI, 2.65–8.20) compared to what their scores would have been had they not been displaced.

We applied the same AIPW and IPWRA approaches to depression symptom scores, with Y(0) and Y(1) representing the depression scores if not displaced or displaced, respectively.

For depression, the POmeans were as follows. Under the non-displacement scenario (Y(0)), the estimated mean depression score was 4.25 (95% CI, 3.90–4.60). In the ATE scenario (Y(1)), where displacement occurs universally, the mean depression score would increase to 6.18 (95% CI, 5.28–7.08), shifting individuals from minimal to mild depression. Among those who were actually displaced, the observed mean depression score (Y(1) | D = 1) was 6.52 (95% CI, 5.50–7.56), while their counterfactual mean had they not been displaced (Y(0) | D = 1) would have been 4.41 (95% CI, 3.82–4.99).

The ATE for depression was estimated at 1.93 points (95% CI, 0.99–2.87), indicating a shift in depression scores if displacement were universal. The ATT for depression was 2.12 points (95% CI, 1.04–3.20), highlighting an increase in depressive symptoms among those who were actually displaced compared to their counterfactual scores had they not been displaced.

Overall, these results suggest that displacement following Hurricane Harvey is associated with higher PTSD and depression symptom scores among affected residents, even after adjusting for a comprehensive set of covariates. The POmeans illustrate the hypothetical shifts in symptom severity under scenarios of no displacement versus universal displacement. The ATE estimates reveal population-level shifts, while the ATT estimates confirm the increased symptom severity experienced by those who were actually displaced.

The estimated POmeans, ATE, and ATT of displacement on PTSD and depression symptom scores are detailed in Table 3, respectively.

**Table 3:** (a) Population-level effects: What if displacement affected everyone vs. no one? (ATE). (b) Individual-level effects: Impact on those who were actually displaced (ATT).

Mental health outcome	No displacement scenario Y(0)	Universal displacement scenario Y(1)	Population effect (95% CI)
(a) Population-level effects: What if displacement affected everyone vs. no one? (ATE).			
PTSD symptoms	10.85 (9.92–11.78)	16.73 (14.35–19.10)	5.88* (3.37–8.39)
Depression symptoms	4.25 (3.90–4.60)	6.18 (5.28–7.08)	1.93* (0.99–2.87)
(b) Individual-level effects: Impact on those who were actually displaced (ATT)			
**Mental health outcome**	**If they had not been displaced Y(0|D = 1)**	**Observed scores Y(1|D = 1)**	**Individual effect (95% CI)**
PTSD symptoms	11.49 (10.01–12.96)	16.91 (14.84–19.00)	5.43* (2.65–8.20)
Depression symptoms	4.41 (3.82–4.99)	6.52 (5.50–7.56)	2.12* (1.04–3.20)

*All effects p < 0.001.

ATE (Average Treatment Effect) represents the estimated population-level contrast under counterfactual scenarios of universal versus no displacement. ATT (Average Treatment Effect on the Treated) represents the estimated contrast specifically for individuals who experienced displacement. Y(0) and Y(1) denote potential outcomes under counterfactual scenarios of no displacement and displacement, respectively. For ATT, Y(0|D = 1) represents counterfactual outcomes for displaced individuals had they not been displaced, and Y(1|D = 1) represents their observed outcomes. CI = confidence interval. PTSD (post-traumatic stress disorder) measured using the PTSD Checklist for DSM-5 (PCL-5); depression measured using the Patient Health Questionnaire-9 (PHQ-9). ATE estimated using augmented inverse probability weighting (AIPW); ATT estimated using inverse probability weighted regression adjustment (IPWRA). Confidence intervals are based on bootstrap standard errors with 1,000 replications.

### Robustness checks and covariate balance

**Fig 1:**
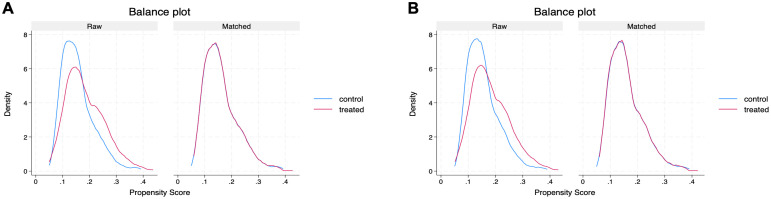
Balance plots showing propensity score distributions from propensity score matching. (A) PTSD model. (B) Depression model. The blue lines represent the density of propensity scores for the control (non-displaced) group; the red lines represent the treated (displaced) group. Before matching (Raw panels), the propensity score distributions of the two groups differ noticeably, indicating a lack of balance. After matching (Matched panels), the distributions overlap substantially, indicating that the matching process successfully balanced the covariates between the groups.

Covariate balance was assessed using standardized mean differences (SMDs), variance ratios, overidentification tests, and propensity score distribution plots. After weighting, all SMDs were well below the acceptable threshold of 0.10, with the largest weighted SMD being 0.060 (Table C in S1 Appendix) [29,30]. Variance ratios were all within the acceptable range of 0.80–1.25 after weighting. The overidentification test for covariate balance yielded a chi-square statistic of 15.23 (p = 0.976) for PTSD and 14.62 (p = 0.982) for depression, indicating that the covariates were adequately balanced after weighting. The resulting balance plots (Fig 1A for PTSD and Fig 1B for depression) show that, before weighting, the propensity score distributions differed clearly between the non-displaced and displaced groups, whereas after weighting, these distributions overlapped substantially for both outcomes, supporting the validity of the AIPW and IPWRA estimations.

Examination of the inverse probability weight distributions revealed no extreme weights. The weight distribution was approximately symmetric (skewness = 0.25), with no observation exceeding 2.8 standard deviations from the mean weight, and a maximum-to-minimum weight ratio of 16.6. For the PTSD model, the effective sample size (ESS) was 800.9 out of a nominal sample of 1,018 (ESS/N ratio = 0.79), indicating that approximately 79% of the sample’s information was retained after weighting (Table D in S1 Appendix).

To verify that the choice of estimator did not influence the conclusions, we estimated all four combinations of estimator (AIPW, IPWRA) and estimand (ATE, ATT). Point estimates were substantively identical across all specifications: for PTSD, the ATE ranged from 5.81 (IPWRA) to 5.88 (AIPW), and the ATT from 5.43 (AIPW) to 5.43 (IPWRA). For depression, the ATE ranged from 1.91 (IPWRA) to 1.93 (AIPW), and the ATT from 2.12 (AIPW) to 2.12 (IPWRA) (Table B in S1 Appendix). This consistency demonstrates that the reported effects are robust to the choice of estimator.

To further assess the robustness of our findings, we conducted post hoc sensitivity analyses. Using the konfound framework [31,32], we found that nullifying the estimated effects would require replacing approximately 50% of observations or an omitted confounder with correlations exceeding 0.25 with both exposure and outcome. Full regression outputs and sensitivity analyses are provided in the supplemental material.

## Discussion

We examined the association between displacement due to Hurricane Harvey and mental health indicators for PTSD and depression symptoms among residents of Houston, Texas, within a causal inference framework. Using the doubly robust AIPW and IPWRA estimators to adjust for potential confounders, we found that displacement was associated with significantly higher PTSD and depression symptom scores. The ATE of displacement was an increase of 5.88 points in PTSD symptom scores and 1.93 points in depression symptom scores. Similarly, among individuals who were actually displaced, the ATT indicated an increase of 5.43 points in PTSD scores and 2.12 points in depression scores compared to what their scores would have been had they not been displaced. These findings suggest that persons who were displaced due to the hurricane experienced substantially higher levels of PTSD and depression symptom scores compared to those who were not displaced. The shifts in mean symptom scores correspond to transitions into higher severity categories, indicating increased psychological distress that may require clinical intervention.

Notably, the ATE and ATT estimates differed slightly across outcomes. For PTSD, the ATE (5.88) was somewhat larger than the ATT (5.43), whereas for depression, the ATE (1.93) was slightly smaller than the ATT (2.12). This pattern may reflect selection heterogeneity: individuals who were displaced may possess characteristics that modestly attenuate the displacement effect on PTSD relative to the general population, while amplifying it for depression. For instance, displaced individuals were more likely to report lower household income, which may interact differently with PTSD and depression pathways. While these differences are small and should be interpreted cautiously, they underscore the value of reporting both population-level and subgroup-specific effects.

Our results are consistent with prior studies that have documented the adverse mental health effects of displacement following natural disasters. For example, research following Hurricanes Katrina and Sandy in the US demonstrated that displaced persons had higher rates of PTSD and depression compared to non-displaced persons [9,14]. Similarly, studies after the 2011 Great East Japan Earthquake reported increased psychological distress among displaced populations [33]. The magnitude of the effects observed in our study aligns with these earlier findings, reinforcing the notion that displacement is a significant risk factor for poor mental health outcomes post-disaster.

Several factors may contribute to the heightened PTSD and depression symptomology observed among displaced persons. Displacement often involves loss of home, personal belongings, and a sense of security, which can lead to feelings of grief, helplessness, and anxiety. Additionally, displacement may disrupt social networks and access to social support, which are critical for coping with stress and trauma [34]. The uncertainty and challenges associated with temporary housing, financial strain, and navigating assistance programs may further exacerbate mental health issues.

A key strength of this study is the use of the AIPW and IPWRA estimators, both of which provide a doubly robust method for estimating causal effects in observational studies. By combining propensity score weighting with outcome regression models, we addressed potential confounding and enhanced the validity of our findings. Comprehensive balance diagnostics—including standardized mean differences, variance ratios, overidentification tests, effective sample size, and weight distribution examination—confirmed that covariates were well balanced after weighting (all weighted SMDs < 0.10; ESS/N ratio = 0.79), supporting the conditional exchangeability assumption that underpins causal inference. Further, the inclusion of a comprehensive set of covariates, such as socioeconomic status, physical health, and living conditions, allowed for thorough adjustment of potential confounders. While the overall response rate was modest (11.4%), it aligns with national mental health survey benchmarks—for example, the 2021–2022 National Survey on Drug Use and Health reported similar rates (12.1% nationally; 11.4% in Texas) [35]. Importantly, causal inference relies primarily on internal validity and conditional exchangeability rather than full population representativeness; our analysis satisfies this requirement through rigorous covariate balance and doubly robust estimation, even if the response rate limits generalizability.

## Limitations

Several limitations should be acknowledged. First, the cross-sectional design precludes establishing temporal causality between displacement and mental health outcomes. Although displacement occurred in 2017 prior to the assessment of mental health indicators—which were measured over the past month in 2020—the simultaneous collection of exposure and outcome data means we cannot entirely rule out the possibility that pre-existing mental health conditions influenced both the likelihood of being displaced and the severity of post-disaster symptom reporting. Moreover, the 3–4 year gap between Hurricane Harvey and data collection creates the potential for time-varying confounding: intervening life events (e.g., job loss, family changes, new traumatic experiences, and the onset of the COVID-19 pandemic) may have concurrently affected mental health status and been associated with displacement status. Covariates such as age, race/ethnicity, and education are time-invariant or pre-determined, but income was measured for 2019 and physical health reflects self-reported status at the time of the survey. We acknowledge that some covariates may reflect post-displacement rather than pre-displacement conditions. Nevertheless, our use of a doubly robust estimator and extensive covariate adjustment should partially mitigate these concerns.

Second, the reliance on self-reported data may introduce reporting bias. Participants might have underreported or overreported their symptoms due to stigma or recall bias. Additionally, displacement status was self-reported, and we did not have objective measures of the severity of home damage or the duration of displacement. Nevertheless, self-reporting is a common and practical method in this context, and we used validated instruments and assured confidentiality to encourage accurate reporting.

Third, by using displacement as a binary exposure, we did not account for the potential dose-response relationship that may exist with varying levels of displacement, such as duration, number of relocations, stability of temporary housing, or separation from family. Treating displacement as a binary variable simplifies the exposure but risks underestimating the true effect if there is heterogeneity in exposure intensity that influences mental health outcomes like PTSD and depression. Although we acknowledge the potential for treatment effect heterogeneity (i.e., different levels of displacement may have different impacts) [36], our binary approach provides a clear, interpretable estimate of the overall effect of displacement on mental health indicators. Future studies should aim to capture these dimensions to better characterize the dose-response relationship between displacement severity and mental health outcomes.

Fourth, despite our efforts to apply a rigorous causal inference framework, unmeasured confounding cannot be entirely ruled out. While we incorporated a comprehensive set of theoretically informed covariates, key potential confounders—including prior trauma history, pre-disaster mental health status, detailed employment and housing insecurity indicators, social support, and severity of disaster exposure—were not available in the dataset. Several of these unmeasured variables are plausibly correlated with both displacement and mental health outcomes, potentially at levels that could influence the reported estimates. Our sensitivity analyses using the konfound framework found that nullifying the observed effects would require an omitted variable with correlations of approximately 0.26 with both exposure and outcome. While this threshold is non-trivial, we acknowledge that variables such as prior trauma exposure or baseline mental health status could plausibly approach or exceed this level of confounding. The doubly robust AIPW and IPWRA estimators provide protection against model misspecification but do not protect against omitted variable bias. Accordingly, while our doubly robust approach and counterfactual framework strengthen inference beyond conventional regression, the estimates should be interpreted as associations given the potential for residual confounding.

Fifth, we did not conduct an a priori sample size estimation, as the study relied on an existing observational survey with a fixed sample. The effective sample size after propensity score weighting (ESS = 800.9) was smaller than the nominal sample, which may affect precision of the estimates, though all primary effects remained statistically significant.

Our findings advance theoretical understandings of disaster-related displacement and trauma by quantifying the association between displacement and mental health outcomes using a counterfactual framework that moves beyond conventional descriptive approaches. This strengthens ecological and stress-process frameworks by distinguishing the potential psychological impact of displacement itself—beyond disaster exposure or sociodemographic risk factors.

The use of counterfactual methods enables us to move beyond descriptive correlations, offering empirical estimates of how displaced individuals would have fared had they not been displaced.

Further, our findings have important implications for disaster preparedness and response efforts. Mental health services should be an integral component of disaster relief programs, with particular attention to persons who experience displacement. Early identification and intervention for those at risk of developing PTSD and depression can help mitigate long-term psychological consequences. Policies aimed at providing stable housing solutions and facilitating the restoration of social networks may also alleviate psychological distress among displaced populations.

Future studies should consider longitudinal designs to examine changes in mental health outcomes over time and establish temporal relationships. Displacement is not a homogenous exposure—its impact likely varies based on factors such as duration, housing stability, social support, and access to resources—underscoring the need for nuanced, context-sensitive research that captures the heterogeneity of displacement experiences. Additionally, qualitative research exploring personal experiences of displacement may enrich our understanding of the specific challenges faced by affected persons.

Displacement due to Hurricane Harvey was associated with increased symptoms of PTSD and depression among residents of Houston. While our doubly robust analytical approach and comprehensive sensitivity analyses strengthen confidence in these findings, the absence of pre-disaster mental health data and other key confounders means the reported estimates are best interpreted as associations that are suggestive of, but not definitive evidence for, a causal effect of displacement. These results underscore the critical need for incorporating mental health support into disaster response initiatives, particularly for displaced persons. Addressing the psychological impact of displacement can contribute to more effective recovery efforts and improve the well-being of affected communities.

## Supporting information

S1 Appendix Supplemental tables. (DOCX)
