# Supplementary material for: Understanding the impact of natural disasters on post-traumatic stress disorder and depression symptoms: An examination of counterfactual displacement scenarios
Source: PLOS Ment Health. 2026 Jun 18;3(6):e0000317. doi: 10.1371/journal.pmen.0000317 (PMC13278393; doi:10.1371/journal.pmen.0000317)
Supplement: S1 Appendix — (DOCX) [file pmen.0000317.s001.docx]

**S1 Appendix**

**Table A. Comparison of Study Sample with Houston Adult Population (ACS 2017)**

| **Variable** | **ACS 2017 (%)** | **ACS 2020**  **(%)** | **Study Sample (Unweighted %)** | **Study Sample (Weighted %)** |
| --- | --- | --- | --- | --- |
| **Gender** |  |  |  |  |
| Male | 49.6 | 49.5 | 35.1 | 48.1 |
| Female | 50.4 | 50.5 | 64.3 | 50.6 |
| Other | - | - | 0.6 | 1.3 |
| **Age Groups** |  |  |  |  |
| 18-24 years | 12.9 | 13.3 | 4.7 | 12.0 |
| 25-34 years | 24.8 | 24.5 | 12.5 | 22.2 |
| 35-44 years | 18.5 | 18.6 | 17.7 | 15.9 |
| 45-54 years | 16.0 | 15.5 | 14.4 | 15.9 |
| 55-64 years | 13.9 | 13.9 | 17.7 | 18.4 |
| 65-74 years | 8.6 | 8.7 | 20.3 | 9.1 |
| 75-84 years | 4.0 | 4.0 | 9.4 | 4.7 |
| 85+ years | 1.6 | 1.6 | 3.2 | 1.9 |
| **Race/Ethnicity** |  |  |  |  |
| White, non-Hispanic | 24.7 | 23.5 | 38.1 | 37.3 |
| Black, non-Hispanic | 22.5 | 23.7 | 25.0 | 13.7 |
| Asian, non-Hispanic | 6.5 | 6.9 | - | - |
| Other/Multiracial, non-Hispanic | 1.7 | 0.4 | 8.2 | 5.7 |
| Hispanic (any race) | 44.6 | 45.5 | 28.7 | 43.3 |

Notes:

ACS 2017 1-Year Estimates, Houston city, Texas (Table DP05 - Demographic Characteristics). All percentages calculated for adults 18 years and older.

ACS 2020 5-Year Estimates, Houston city, Texas (Table DP05 - Demographic Characteristics). All percentages calculated for adults 18 years and older.

**Table B.** Cross-estimator consistency: Treatment effect estimates across all estimator–estimand combinations

| **Outcome** | **AIPW-ATE** | **IPWRA-ATE** | **AIPW-ATT** | **IPWRA-ATT** |
| --- | --- | --- | --- | --- |
| PTSD symptoms | 5.88 (3.37, 8.39) | 5.81 (3.36, 8.26) | 5.43 (2.65, 8.20) | 5.43 (2.66, 8.20) |
| Depression symptoms | 1.93 (0.99, 2.87) | 1.91 (0.99, 2.83) | 2.12 (1.04, 3.20) | 2.12 (1.05, 3.19) |

**Note:** AIPW = Augmented Inverse Probability Weighting; IPWRA = Inverse Probability Weighted Regression Adjustment; ATE = Average Treatment Effect; ATT = Average Treatment Effect on the Treated. PTSD measured using the PTSD Checklist for DSM-5 (PCL-5; range 0–80). Depression measured using the Patient Health Questionnaire-9 (PHQ-9; range 0–27). Values are point estimates (95% confidence intervals from bootstrap standard errors with 1,000 replications). All effects p < 0.001.

**Table C.** Covariate balance before and after inverse probability weighting (PTSD model)

| **Covariate** | **Raw SMD** | **Weighted SMD** | **Raw VR** | **Weighted VR** |
| --- | --- | --- | --- | --- |
| Female (vs. Male) | 0.085 | 0.019 | 0.953 | 0.989 |
| Other (vs. Male) | −0.011 | −0.008 | 0.879 | 0.906 |
| Age 25–34 (vs. 18–24) | −0.106 | −0.003 | 0.776 | 0.993 |
| Age 35–44 | 0.047 | 0.007 | 1.085 | 1.012 |
| Age 45–54 | 0.040 | −0.060 | 1.087 | 0.880 |
| Age 55–64 | −0.092 | 0.015 | 0.850 | 1.026 |
| Age 65–74 | −0.015 | −0.009 | 0.982 | 0.987 |
| Age 75–84 | 0.136 | 0.020 | 1.427 | 1.056 |
| Age 85+ | 0.080 | 0.029 | 1.514 | 1.161 |
| HS graduate/GED (vs. <HS) | 0.005 | −0.016 | 1.014 | 0.971 |
| Some college/technical | 0.140 | −0.010 | 1.163 | 0.988 |
| College/graduate degree | −0.162 | 0.030 | 0.985 | 0.999 |
| Black, non-Hispanic (vs. White) | 0.126 | 0.032 | 1.149 | 1.036 |
| Other/2+ races, non-Hispanic | 0.032 | −0.020 | 1.103 | 0.940 |
| Hispanic | 0.007 | −0.005 | 1.011 | 0.996 |
| Never married (vs. Div/Sep/Wid) | −0.043 | 0.028 | 0.962 | 1.027 |
| Married | −0.074 | −0.023 | 0.984 | 0.994 |
| $25,000–$49,999 (vs. ≤$24,999) | −0.109 | −0.023 | 0.857 | 0.968 |
| $50,000–$74,999 | −0.107 | 0.025 | 0.791 | 1.052 |
| $75,000–$99,999 | 0.040 | −0.020 | 1.114 | 0.946 |
| $100,000–$149,999 | −0.099 | 0.020 | 0.761 | 1.051 |
| $150,000–$199,999 | −0.139 | 0.018 | 0.563 | 1.067 |
| ≥$200,000 | −0.025 | 0.005 | 0.950 | 1.012 |
| Rent (vs. Own) | 0.063 | 0.010 | 1.045 | 1.006 |
| Living with family | 0.040 | −0.014 | 1.310 | 0.903 |
| Other living | 0.005 | 0.006 | 1.054 | 1.055 |
| Physical health (ordinal) | 0.153 | −0.035 | 1.179 | 1.204 |

**Note:** SMD = standardized mean difference; VR = variance ratio. Adequate balance indicated by |SMD| < 0.10 and VR between 0.80 and 1.25 after weighting. All weighted SMDs are below the 0.10 threshold. Results from the depression model are substantively identical and available upon request. Raw: before propensity score weighting. Weighted: after propensity score weighting.

**Table D.** Inverse probability weight diagnostics and effective sample size

| **Diagnostic** | **PTSD Model** | **Depression Model** |
| --- | --- | --- |
| Nominal sample size (N) | 1,018 | 1,015 |
| Effective sample size (ESS) | 800.9 | —* |
| ESS / N ratio | 0.79 | —* |
|  |  |  |
| IPW weight: mean (standard deviation) | 6.37 (3.32) | —* |
| IPW weight: median | 6.38 | —* |
| IPW weight: minimum | 1.07 | —* |
| IPW weight: 1st percentile | 1.10 | —* |
| IPW weight: 99th percentile | 14.56 | —* |
| IPW weight: maximum | 17.82 | —* |
| IPW weight: max/min ratio | 16.6 | —* |
|  |  |  |
| Overidentification χ² (p-value) | 15.23 (0.976) | 14.62 (0.982) |
| Weighted treated obs | 510.3 | 508.5 |
| Weighted control obs | 507.7 | 506.5 |

**Note:** ESS = effective sample size, computed using Kish’s formula: ESS = (∑w)² / ∑w². IPW = inverse probability weight. ESS/N ratio > 0.80 indicates minimal information loss from weighting. Max/min weight ratio < 20 indicates no extreme weights. *Depression model diagnostics are substantively identical to the PTSD model (same propensity score specification); overidentification test statistics are reported for both outcomes.
