# Supplementary material for: Understanding the impact of natural disasters on post-traumatic stress disorder and depression symptoms: An examination of counterfactual displacement scenarios
Source: PLOS Ment Health. 2026 Jun 18;3(6):e0000317. doi: 10.1371/journal.pmen.0000317 (PMC13278393; doi:10.1371/journal.pmen.0000317)
Supplement: Attachment — Submitted filename: Response to Reviewers_PLOS_MH.docx [file pmen.0000317.s002.docx]

**Response to Editors Comments**

**Journal Requirements:**
1. Please provide additional details regarding participant consent. In the ethics statement in the Methods and online submission information, please ensure that you have specified (1) whether consent was informed and (2) what type you obtained (for instance, written or verbal, and if verbal, how it was documented and witnessed). If your study included minors, state whether you obtained consent from parents or guardians. If the need for consent was waived by the ethics committee, please include this information.

If you are reporting a retrospective study of medical records or archived samples, please ensure that you have discussed whether all data were fully anonymized before you accessed them and/or whether the IRB or ethics committee waived the requirement for informed consent. If patients provided informed written consent to have data from their medical records used in research, please include this information.

1) Yes consent was informed. 2) Consent was provided when individuals agreed to participate and completed and returned the study survey. We obtained a waiver of documentation of consent, because the research presented no more than minimal risk of harm to the subjects and involved no procedures for which written consent would normally be required outside of the research

2. Please insert an Ethics Statement at the beginning of your Methods section, under a subheading 'Ethics Statement'. It must include:

1) The name(s) of the Institutional Review Board(s) or Ethics Committee(s)

2) The approval number(s), or a statement that approval was granted by the named board(s)

3) (for human participants/donors) - A statement that formal consent was obtained (must state whether verbal/written) OR the reason consent was not obtained (e.g. anonymity). NOTE: If child participants, the statement must declare that formal consent was obtained from the parent/guardian.

Thank you for this clarification. We have inserted a dedicated “Ethics Statement” at the beginning of the Methods section in the revised manuscript. The updated statement also includes the name of the Institutional Review Board (Boston University Medical Campus IRB) and the approval number (H-39516)

3. Please provide separate figure files in .tif or .eps format.

Thank you for this instruction. We have provided separate figure files in the required formats (.tif/.eps) as part of our revised submission. Please let us know if any additional formatting adjustments are needed.

For more information about figure files please see our guidelines:

[https://journals.plos.org/mentalhealth/s/figures](https://nam10.safelinks.protection.outlook.com/?url=https%3A%2F%2Fjournals.plos.org%2Fmentalhealth%2Fs%2Ffigures&data=05%7C02%7Cabba-aji%40wustl.edu%7C475e24c381af4a0fb34708dd8caa45ba%7C4ccca3b571cd4e6d974b4d9beb96c6d6%7C0%7C0%7C638821385166008688%7CUnknown%7CTWFpbGZsb3d8eyJFbXB0eU1hcGkiOnRydWUsIlYiOiIwLjAuMDAwMCIsIlAiOiJXaW4zMiIsIkFOIjoiTWFpbCIsIldUIjoyfQ%3D%3D%7C0%7C%7C%7C&sdata=LJlYiAN1XZtLpSHJcPv%2FtCsO2X%2B8sQVVMN4UBtT1zmc%3D&reserved=0) 
[https://journals.plos.org/mentalhealth/s/figures#loc-file-requirements](https://nam10.safelinks.protection.outlook.com/?url=https%3A%2F%2Fjournals.plos.org%2Fmentalhealth%2Fs%2Ffigures%23loc-file-requirements&data=05%7C02%7Cabba-aji%40wustl.edu%7C475e24c381af4a0fb34708dd8caa45ba%7C4ccca3b571cd4e6d974b4d9beb96c6d6%7C0%7C0%7C638821385166023287%7CUnknown%7CTWFpbGZsb3d8eyJFbXB0eU1hcGkiOnRydWUsIlYiOiIwLjAuMDAwMCIsIlAiOiJXaW4zMiIsIkFOIjoiTWFpbCIsIldUIjoyfQ%3D%3D%7C0%7C%7C%7C&sdata=69yhEYlJiGOAHX7dRN%2FHLrfP4CBYPCiA1yo8sDqnsUM%3D&reserved=0)

4. In the online submission form, you indicated that “Data is available upon request.”.

All PLOS journals now require all data underlying the findings described in their manuscript to be freely available to other researchers, either 
1. In a public repository, 
2. Within the manuscript itself, or 
3. Uploaded as supplementary information.

This policy applies to all data except where public deposition would breach compliance with the protocol approved by your research ethics board. If your data cannot be made publicly available for ethical or legal reasons (e.g., public availability would compromise patient privacy), please explain your reasons by return email and your exemption request will be escalated to the editor for approval. Your exemption request will be handled independently and will not hold up the peer review process, but will need to be resolved should your manuscript be accepted for publication. One of the Editorial team will then be in touch if there are any issues.

Regarding data availability, we would like to share the data used in this manuscript. However, in consultation with our Institutional Review Board at Boston University School of Medicine, we have determined that we cannot share the entire data set used in this analysis, outside our study team, even in deidentified form. Our study protocol included a data sharing consent form, per guidelines of the National Institute of Mental Health, and approximately 80% of the people in our study sample declined to provide permission to share their deidentified data. Thus, we could only feasibly share data outside of our study team from about 20% of participants, which would not allow study replication. The point of contact to the Institutional review board at Boston University Medical Campus is the email: medirb@bu.edu.

**Response to Reviewer’s Comments**

Reviewer #1: The authors examined the cross-sectional association between residential relocation and trauma-related psychopathology. While they offered valuable insight on the risk of PTSD/depression after displacement by considering the counterfactual scenario, the low response rate (approximately 10%) limits the confidence in the representativeness of the target population and raises concerns about selection bias.

We thank the reviewer for recognizing the value of our approach in examining the mental health impact of displacement through counterfactual modeling. We appreciate this encouragement and have worked to address the noted concerns regarding representativeness and selection bias, as detailed below.

1. Can you show a table comparing key demographic and clinical characteristics of the target population and your analytic sample? It might help readers understand what population you analyzed.

We thank the reviewer for this suggestion. We have added text (lines 199-202) comparing our sample demographics with the adult Houston population and included a supplementary table (Table S1) with detailed demographic comparisons using data from the American Community Survey (ACS) 2017 and 2020. This comparison demonstrates the representativeness of our sample and supports readers in assessing the generalizability of our findings.
2. Weighting adjustment is necessary to mitigate the estimation bias because of the low response rate.

We thank the reviewer for highlighting the importance of weighting adjustments for addressing low response rates. We had incorporated complex survey weights into our analysis, and in response to this comment, we have enhanced our reporting of this methodology in the manuscript (lines 98–102): "We created complex survey weights to account for differential probabilities of selection, survey response rates, neighborhood size, and household composition. We then post-stratified these weights to align with the demographic distributions of sex, race, ethnicity, age, and educational attainment in the Houston Metropolitan Area, ensuring representativeness of the target population."

Our analytic approach employed Augmented Inverse Probability Weighting (AIPW), which combines the survey weights described above with propensity score-based inverse probability weights. While survey weights ensure external validity by adjusting for sampling design and non-response, the propensity score weights in AIPW address internal validity by reweighting observations to balance covariate distributions between displaced and non-displaced groups, thereby removing confounding bias for causal identification. To further enhance the precision and robustness of our estimates, we employed bias-corrected bootstrapping with 1,000 replications to derive standard errors and confidence intervals. These methodological choices, combined with our weighting strategy, strengthen the validity of our causal inferences despite the challenges posed by the low response rate typical of post-disaster research.

3. Please justify how your results advance or refine existing theories of disaster‑related displacement and trauma, despite the remaining methodological limitations (low response rate).

We thank the reviewer for the prompt. In response, we have now incorporated a new passage into the Discussion section (lines 341–346) that articulates how our findings advance theoretical understandings of disaster-related displacement and trauma. Specifically, the revised text highlights that our study quantifies displacement as an independent causal stressor, thereby strengthening ecological and stress-process frameworks by isolating the psychological impact of displacement itself. This addition underscores how our use of counterfactual methods moves beyond descriptive correlations, providing empirical estimates of how displaced individuals would have fared had they not been displaced.

Thank you again for these helpful reviews and the opportunity to revise and improve our manuscript. Should you have any further concerns or revisions you would like us to make please do not hesitate to let us know.

Warmly,

Mohammed Abba-Aji
