# Supplementary material for: Understanding the impact of natural disasters on post-traumatic stress disorder and depression symptoms: An examination of counterfactual displacement scenarios
Source: PLOS Ment Health. 2026 Jun 18;3(6):e0000317. doi: 10.1371/journal.pmen.0000317 (PMC13278393; doi:10.1371/journal.pmen.0000317)
Supplement: Attachment — Submitted filename: Response to Editor_Reviewer _PLOS_MH_2.docx [file pmen.0000317.s003.docx]

Mohammed Abba-Aji

Department of Epidemiology

715 Albany St.

Boston, MA 02118

Oct 19, 2025

Dear Editor,

**Resubmission following appeal**

Thank you for your kind response and for granting us the opportunity to revise and resubmit our manuscript, “***Understanding the impact of natural disasters on post-traumatic stress disorder and depression symptoms: An examination of counterfactual displacement scenarios***” which appears to rest primarily on concerns regarding sample representativeness given a response rate of 11.4%.

We understand and acknowledge the reviewer’s concerns regarding this response rate and the potential for selection bias. However, it is important to note that our response rate is commensurate with national benchmarks for large-scale mental health surveys. For instance, the 2021–2022 National Survey on Drug Use and Health (NSDUH)—the largest federally funded mental health survey in the U.S.—reported overall response rates of 12.1% nationally and 11.4% in Texas during the same period as our study ([Table C1](https://www.samhsa.gov/data/sites/default/files/reports/rpt44483/2022-nsduh-sae-guide-state-meth/2022-nsduh-sae-guide-state-meth.htm#tabc-1)). We have further expanded our methodological discussion and added a new supplemental section detailing sensitivity analyses that further strengthen the robustness of our findings.

We have acknowledged the reviewer’s concern and have addressed it with transparency and rigor throughout our analysis. Below, we note the reviewer’s concern and clarify why the core conclusions of our study remain valid:

**Reviewer Concern:**
The study’s low response rate and persistent racial imbalance—even after weighting—undermine the reliability of its conclusions.

**Our Response :**

1. **Explicitly acknowledged and addressed response rate limitations** – We are transparent in the manuscript about the limitations posed by response rate. This limitation is standard in post-disaster survey research, where barriers to participation are often heightened. In fact, low response is a recognized and commonly noted challenge in the disaster literature. (lines 321-326)
2. **Distinction between internal and external validity** – The reviewer’s concern centers on generalizability. However, causal inference relies primarily on internal validity and exchangeability conditional on covariates—not full population representativeness. Our analysis meets that threshold, offering robust estimates of the causal impact of displacement on mental health.
3. **Applied population weights and bootstrapped estimates** – We incorporated post-stratification weights and used 1,000-replication bootstrapping to generate robust standard errors. These additional safeguards bolster the stability and reliability of our findings despite the modest response rate.
4. **Well Balanced groups** – We further note that, beyond applying survey weighting to address demographic representativeness, we rigorously evaluated covariate balance using an overidentification test. The test yielded a chi-square statistic of 14.98 (*p* = 0.9576) for PTSD and 14.37 (*p* = 0.968) for depression, indicating that the covariates were adequately balanced after weighting. As shown in the balance plots (Fig. 1a–b), prior to weighting, the propensity score distributions differed substantially between treated and control groups; after weighting, these distributions overlapped almost entirely for both outcomes. Given this high degree of covariate balance, the potential for systematic bias is substantially mitigated, even in the presence of a modest survey response rate.

**Fig. 1 a. Balance plot for PTSD score	Fig. 1 b. Balance plot for depression score**

5. **Used AIPW and IPWRA estimation** – Our analytic approach centers on Augmented Inverse Probability Weighting (AIPW) and Inverse Probability Weighted Regression Adjustment (IPWRA), doubly robust estimators that mitigate bias due to both treatment assignment and outcome model misspecification. These are widely accepted approaches in causal inference for improving internal validity when complete representativeness is not feasible.
6. **Selection Bias and Unmeasured Confounding-** To assess the impact of unmeasured confounding we incorporate new **sensitivity analyses using the konfound framework.** These analyses quantify how much unmeasured confounding would be required to overturn our main findings. Results show that nullifying the estimated effects would require replacing approximately **50% of observations** with cases showing no effect (RIR ≈ 506–514), and that an omitted variable would need correlations of **r ≈ 0.25 with both the outcome and predictor** to do so. These results are now summarized in the **Discussion (lines 281-286)** and presented in detail in the **Supplementary Materials**
7. **The critique does not invalidate the central findings** – Our study quantifies the mental health burden of displacement using counterfactual scenarios, a methodological advance over prior descriptive studies. We understand that the reviewer does not contest the correctness of our estimation strategy—only the sample representativeness, which we addressed within the bounds of methodological best practices.

In light of these considerations, we respectfully request reconsideration of our manuscript. We are open to additional clarifications or improvements as needed and are committed to ensuring the highest standard of transparency and methodological integrity.

Thank you for your time and thoughtful review.

Sincerely,
Mohammed Abba-Aji, MD, MPH
