# Supplementary material for: Understanding the impact of natural disasters on post-traumatic stress disorder and depression symptoms: An examination of counterfactual displacement scenarios
Source: PLOS Ment Health. 2026 Jun 18;3(6):e0000317. doi: 10.1371/journal.pmen.0000317 (PMC13278393; doi:10.1371/journal.pmen.0000317)
Supplement: Attachment — Submitted filename: PMEN_R2_Response_to_Reviewers_FINAL.docx [file pmen.0000317.s004.docx]

We are grateful to the reviewers for their encouraging comments and thoughtful engagement with our work. We address all points raised in this revision below. Our responses in are in blue ink and additions to the manuscript are indicated in quotations within our responses.

We believe these revisions have substantially strengthened the manuscript’s methodological rigor and transparency. We remain open to any further suggestions.

# Response to Reviewer #2

**Reviewer #2 stated:** “All comments have been addressed.” We thank Reviewer #2 for this assessment and for their constructive suggestions in this round, which we address below.

**Comment 1: Cross-sectional design and time lag**

The authors correctly acknowledge the limitations of the cross-sectional design in the discussion. However, this issue warrants deeper consideration. Hurricane Harvey occurred in 2017, while the mental health data were collected in 2020-2021, creating a 3-4 year gap... This represents a potential source of unmeasured time-varying confounding. It is recommended that the authors more explicitly highlight this point in the limitations section of the discussion.

**Response:** We agree that the 3–4 year gap between Hurricane Harvey and data collection warrants deeper consideration. We have substantially expanded Limitation 1 in the Discussion (lines 366–373) to explicitly address time-varying confounding. The revised text now reads:

*“Moreover, the 3–4 year gap between Hurricane Harvey and data collection creates the potential for time-varying confounding: intervening life events (e.g., job loss, family changes, new traumatic experiences, and the onset of the COVID-19 pandemic) may have concurrently affected mental health status and been associated with displacement status. Covariates such as age, race/ethnicity, and education are time-invariant or pre-determined, but income was measured for 2019 and physical health reflects self-reported status at the time of the survey. We acknowledge that some covariates may reflect post-displacement rather than pre-displacement conditions.”*

**Comment 2: Definition and measurement of exposure**

Operationalizing "forced displacement" as a binary variable (yes/no) is clear but simplifies a complex reality... Dimensions such as the duration of displacement, the number of relocations, the stability of temporary housing, and separation from family could potentially constitute a dose-response relationship... emphasizing the importance of these dimensions in the discussion and suggesting them for future research would make the paper more forward-looking.

**Response:** We have expanded Limitation 3 (lines 382–390) to explicitly name the displacement dimensions suggested by the reviewer and frame them as priorities for future research. The revised text now includes:

*“Third, by using displacement as a binary exposure, we did not account for the potential dose-response relationship that may exist with varying levels of displacement, such as duration, number of relocations, stability of temporary housing, or separation from family... Future studies should aim to capture these dimensions to better characterize the dose-response relationship between displacement severity and mental health outcomes.”*

**Comment 3: Sample representativeness and response rate**

The authors have provided a very thorough and professional defense regarding this issue in their response to the editor... I would recommend that the core arguments from the response letter—specifically the comparison with NSDUH, the primacy of internal validity, and the efforts to address concerns via weighting and sensitivity analyses—be concisely integrated into the Methods section or the limitations paragraph of the manuscript’s Discussion.

**Response:** We agree that these arguments should be self-contained within the manuscript. We have integrated them into the strengths paragraph of the Discussion (lines 353–359), which now includes:

*“While the overall response rate was modest (11.4%), it aligns with national mental health survey benchmarks—for example, the 2021–2022 National Survey on Drug Use and Health reported similar rates (12.1% nationally; 11.4% in Texas). Importantly, causal inference relies primarily on internal validity and conditional exchangeability rather than full population representativeness; our analysis satisfies this requirement through rigorous covariate balance and doubly robust estimation, even if the response rate limits generalizability.”*

**Comment 4: Table labeling error**

In the results section, the titles for Table 3a and Table 3b are duplicated. The first table (ATE) is titled "Table 3a. Population-level effects...", while the second table (ATT) is also titled "Table 3a. Individual-level effects...". This appears to be a typesetting error.

**Response:** Thank you for catching this error. The second table (line 273) has been corrected to “Table 3b. Individual-level effects: Impact on those who were actually displaced (ATT).”

**Comment 5: Interpretation of ATE and ATT results**

I would suggest that the discussion could briefly compare and interpret the difference between the ATE and ATT estimates. For instance, for PTSD, the ATE (5.88) is slightly larger than the ATT (5.43), whereas for depression, the ATE (1.93) is slightly smaller than the ATT (2.12). What might these slight differences suggest?

**Response:** We appreciate this insightful suggestion. We have added a new paragraph to the Discussion (lines 320–328) interpreting the ATE–ATT divergence:

*“Notably, the ATE and ATT estimates differed slightly across outcomes. For PTSD, the ATE (5.88) was somewhat larger than the ATT (5.43), whereas for depression, the ATE (1.93) was slightly smaller than the ATT (2.12). This pattern may reflect selection heterogeneity: individuals who were displaced may possess characteristics that modestly attenuate the displacement effect on PTSD relative to the general population, while amplifying it for depression. For instance, displaced individuals were more likely to report lower household income, which may interact differently with PTSD and depression pathways. While these differences are small and should be interpreted cautiously, they underscore the value of reporting both population-level and subgroup-specific effects.”*

**Comment 6: Handling of missing data**

For the sake of absolute clarity, it is recommended that the Methods section explicitly state that all primary analyses (AIPW/IPWRA) were run on each of the 15 imputed datasets separately, with the results then pooled (e.g., using Rubin’s rules).

**Response:** We have clarified the missing data handling in the Methods section (lines 171–173). The revised text now reads:

*“Missing data were minimal (<5% for all variables). Missing values were addressed through imputation in a preprocessing step prior to analysis; the imputation model included all variables used in the analysis.”*

# Response to Reviewer #3

**Reviewer #3 stated:** “All comments have been addressed.” We thank Reviewer #3 for their continued support of the manuscript.

**Comment: Abbreviations in tables**

The remaining issue to be resolved is that all abbreviations (e.g., IQR, CI, etc.) should be spelled out in full not only in the main text but also in all tables.

**Response:** We have expanded all abbreviations in the table footnotes throughout the manuscript. Table 2 now defines IQR (interquartile range), PCL-5 (PTSD Checklist for DSM-5), and PHQ-9 (Patient Health Questionnaire-9). Tables 3a and 3b now define ATE (Average Treatment Effect), ATT (Average Treatment Effect on the Treated), CI (confidence interval), AIPW (Augmented Inverse Probability Weighting), IPWRA (Inverse Probability Weighted Regression Adjustment), PCL-5, and PHQ-9. The new Supplementary Tables S2–S4 also include full abbreviation definitions in their footnotes.

# Response to Reviewer #4

We are pleased to hear that Reviewer #4 finds our paper intellectually engaging and timely. We also thank them for their detailed and rigorous critique. Their comments have led to substantial improvements in the transparency and precision of our manuscript. We address each point below.

**Major Comment 1: Unmeasured confounding and causal language (line 355)**

The set of covariates used to adjust for confounding... is rather limited for causal inference in the context of disaster-related mental health. Critically, key potential confounders such as prior trauma history, pre-disaster mental health status, detailed socioeconomic indicators (e.g., employment, housing insecurity), social support, and severity of disaster exposure are not included...

The authors argue that unmeasured confounding is unlikely based on konfound sensitivity analyses and the use of doubly robust estimators. However, this conclusion appears overstated... Moreover, double robustness addresses model misspecification but does not protect against omitted variable bias.

Accordingly, this issue should be treated as a key limitation of the study, and the authors are encouraged to substantially tone down their claims in this section.

**Response:** We accept this critique and have made substantial revisions throughout the manuscript. The estimates are now framed as associations, with the causal interpretation described as suggestive rather than definitive. The changes span four areas:

**First,** we have substantially rewritten Limitation 4 (lines 394–407) to explicitly name the missing confounders identified by the reviewer, acknowledge that the konfound threshold could plausibly be met, and state clearly that the estimates should be interpreted as associations:

*“Key potential confounders—including prior trauma history, pre-disaster mental health status, detailed employment and housing insecurity indicators, social support, and severity of disaster exposure—were not available in the dataset. Several of these unmeasured variables are plausibly correlated with both displacement and mental health outcomes, potentially at levels that could influence the reported estimates... We acknowledge that variables such as prior trauma exposure or baseline mental health status could plausibly approach or exceed this level of confounding. The doubly robust AIPW and IPWRA estimators provide protection against model misspecification but do not protect against omitted variable bias. Accordingly, while our doubly robust approach and counterfactual framework strengthen inference beyond conventional regression, the estimates should be interpreted as associations given the potential for residual confounding.”*

**Second,** we have recalibrated the framing throughout the manuscript. Specific changes appear in the Abstract (line 18), Methods (line 166), Results (line 291), and Discussion (lines 341, 462, 489). Key references to “effects” and “impact” in the context of our findings have been replaced with “associations.” The Abstract now states: “our analysis suggests that displacement is associated with worsened PTSD and depression symptomatology. While our doubly robust approach strengthens inference, these estimates should be interpreted as associations given the potential for residual confounding from unmeasured variables.” The Results summary now reads: “these results suggest that displacement following Hurricane Harvey is associated with higher PTSD and depression symptom scores.” The Discussion opening reads “we examined the association between displacement…within a causal inference framework.”

**Third,** while we retain the counterfactual framework as the analytical approach—since AIPW and IPWRA are designed for this purpose—we now explicitly frame our estimates as associations throughout, acknowledging that a causal interpretation is suggestive rather than definitive given the potential for unmeasured confounding from variables such as prior trauma history and baseline mental health status.

**Fourth,** the conclusion of the manuscript (lines 435–439) now reads: “*While our doubly robust analytical approach and comprehensive sensitivity analyses strengthen confidence in these findings, the absence of pre-disaster mental health data and other key confounders means the reported estimates are best interpreted as associations that are suggestive of, but not definitive evidence for, a causal effect of displacement.*”

We note that the Introduction (lines 74–77) retains language describing the motivation for applying causal inference methods (e.g., “there is a need to use causal inference methods to estimate the impact of displacement”), as this describes the analytical approach and the gap in the literature rather than the interpretation of our results.

**Major Comment 2: AIPW and IPWRA estimand pairing (lines 163, 272)**

2-1. The description of AIPW and IPWRA is incomplete and partially inaccurate. The authors incorrectly imply that IPWRA estimates the ATT specifically, although both AIPW and IPWRA can target either the ATE or ATT depending on the estimand defined...

2-2. Although both AIPW and IPWRA can estimate either ATE or ATT depending on the specification, the manuscript assigns ATE estimation exclusively to AIPW and ATT estimation exclusively to IPWRA without providing methodological justification... The authors should clarify why different estimators were used for different estimands, or alternatively, provide results showing that both AIPW and IPWRA yield consistent estimates of both ATE and ATT.

**Response:** The reviewer is correct that neither AIPW nor IPWRA is inherently tied to a specific estimand. We have addressed this in two ways.

**First,** we revised the Methods (lines 163–168) to clarify that both estimators can target either estimand:

*“Although both AIPW and IPWRA can estimate either the Average Treatment Effect (ATE) or the Average Treatment Effect on the Treated (ATT), we report AIPW for the ATE and IPWRA for the ATT as our primary specifications. To verify that this choice does not influence the conclusions, we estimated all four estimator–estimand combinations (AIPW-ATE, AIPW-ATT, IPWRA-ATE, IPWRA-ATT) and confirmed that both estimators yield substantively identical results for both estimands (Supplementary Table S2).”*

**Second,** we ran all four estimator×estimand combinations and report the results in the manuscript (lines 292–297) and Supplementary Table S2. The results demonstrate near-perfect consistency:

|  | **AIPW-ATE** | **IPWRA-ATE** | **AIPW-ATT** | **IPWRA-ATT** |
| --- | --- | --- | --- | --- |
| PTSD | 5.88 | 5.81 | 5.43 | 5.43 |
| Depression | 1.93 | 1.91 | 2.12 | 2.12 |

The maximum divergence between estimators for the same estimand is 0.07 points (PTSD ATE). This confirms that the choice of estimator is immaterial to the conclusions. Full results with confidence intervals are presented in Supplementary Table S2.

**Major Comment 3: Balance and robustness diagnostics (line 274)**

The balance and robustness section provides limited evidence of adequate covariate balance. Reliance solely on an overidentification test and PS distribution plots is insufficient, as these do not substitute for standardized mean differences (SMDs), effective sample size after weighting, or examination of extreme weights. The extremely high p-values (>0.95) from the χ² tests should be interpreted cautiously... Additionally, konfound results do not address key omitted confounders...

**Response:** We agree that the original balance diagnostics were insufficient. We have substantially expanded the robustness assessment with three new sets of diagnostics:

**1. Standardized mean differences (Supplementary Table S3; reported in manuscript lines 307–313):** We computed SMDs for all 27 covariate indicators before and after weighting. After weighting, all SMDs are well below the conventional threshold of |0.10|, with the largest being 0.060 (age 45–54). All variance ratios fall within or near the 0.80–1.25 acceptable range after weighting.

**2. Weight diagnostics (Supplementary Table S4; reported in manuscript lines 287–291):** The effective sample size (ESS) was 800.9 out of N = 1,018 (ESS/N ratio = 0.79), indicating that approximately 79% of the sample’s information was retained after weighting. The maximum-to-minimum weight ratio was 16.6 Suggesting no evidence of extreme weights driving the estimates.

**3. Cross-estimator consistency (Supplementary Table S2; reported in manuscript lines 293–299):** As described above, all four estimator×estimand combinations produce near-identical results, providing additional evidence of robustness.

We retain the overidentification test and balance plots as complementary diagnostics. The revised Methods (lines 203–214) and Results (lines 275–304) sections now describe all four diagnostic approaches and reference the new supplementary tables.

**Major Comment 4: Post hoc power analysis (line 137)**

The current sample size and power subsection relies on a post hoc power calculation, which offers limited inferential value and may be misleading... Rather than including this calculation, it would be more appropriate to remove this subsection and acknowledge the inability to conduct an a priori sample size estimation... as a study limitation.

**Response:** We agree. The post hoc power analysis subsection has been removed entirely. In its place, we have added a new limitation (Limitation 5) in the Discussion (lines 408–411):

*“Fifth, we did not conduct an a priori sample size estimation, as the study relied on an existing observational survey with a fixed sample. The effective sample size after propensity score weighting (ESS = 800.9) was smaller than the nominal sample, which may affect precision of the estimates, though all primary effects remained statistically significant.”*

**Minor Comment 1: Weighting procedure details (line 98)**

The description of the weighting procedure lacks essential methodological details required for reproducibility and assessment of potential bias. Although the authors state that complex survey weights and post-stratification were applied, the specific steps, models, calibration methods, and diagnostics are insufficiently reported.

**Response:** We have expanded the weighting description in the Methods section (lines 102–114) to detail the three-stage process and to clarify the distinction between survey weights and treatment effect weights:

*“Survey weights were constructed in three stages to ensure representativeness of the target population. First, base weights were computed as the inverse of each address’s selection probability, adjusted for household composition. Second, nonresponse adjustment was applied to account for differential survey response rates across neighborhoods. Third, weights were calibrated via iterative proportional fitting (raking) to align marginal distributions of sex, race/ethnicity, age group, and educational attainment with American Community Survey estimates for the Houston Metropolitan Statistical Area. These survey weights were applied to all descriptive analyses (Tables 1, 2, and S1). For treatment effect estimation, the AIPW and IPWRA estimators construct their own inverse probability weights from a propensity score model that adjusts for the same demographic and socioeconomic factors targeted by the survey weights, plus additional covariates. This approach provides doubly robust estimates that account for confounding through both the propensity score and outcome regression components.”*

**Minor Comment 2: “Treatment group” terminology (line 112)**

Referring to displaced individuals as a ‘treatment group’ is conceptually inappropriate, as displacement is not an intervention but an externally imposed exposure. This terminology may mislead readers by implying an experimental design. I recommend renaming this group as the ‘exposed group’ or ‘displaced group’ to maintain clarity and conceptual accuracy.

**Response:** We agree. All instances of “treatment group” and “comparison group” have been replaced with “displaced group” and “non-displaced group,” respectively, throughout the manuscript. The treatment indicator has been relabeled as the “exposure indicator” in the estimand notation section.

**Minor Comment 3: Temporal sequence claim (line 34)**

The authors state that displacement-related variables and covariates represent conditions prior to or at the time of displacement. However, because exposure and covariates were collected concurrently in 2020, the manuscript does not clearly explain how pre-displacement status was established... The authors should clarify how recall bias, post-displacement changes, or time-varying confounders were addressed.

**Response:** We have revised the first limitation in the Discussion (lines 370–374) to be more transparent about what the temporal sequence can and cannot establish. The revised text now explicitly acknowledges:

*“Covariates such as age, race/ethnicity, and education are time-invariant or pre-determined, but income was measured for 2019 and physical health reflects self-reported status at the time of the survey. We acknowledge that some covariates may reflect post-displacement rather than pre-displacement conditions.”*

The revised manuscript no longer claims that temporal ordering was “carefully ensured” for all variables, but rather specifies which covariates are time-invariant and which may be time-varying.

**Minor Comment 4: Balance diagnostics (line 186)**

The overidentification test alone does not provide sufficient evidence of covariate balance. Balance evaluation typically relies on standardized mean differences (SMDs), effective sample size (ESS), and examination of weight distributions. Without these diagnostics, the claim of ‘adequate balance’ is not fully supported.

**Response:** We have addressed this comprehensively, as detailed in our response to Major Comment 3 above. The revised manuscript now reports four complementary balance diagnostics (Methods lines 203–214; Results lines 275–304): (1) standardized mean differences for all covariates (Supplementary Table S3), (2) the overidentification test, (3) propensity score distribution plots, and (4) effective sample size and weight distribution examination (Supplementary Table S4). The Methods section has been updated to describe all four approaches, and the Results section reports the findings from each.

# Summary of Changes

For the editor’s convenience, we summarize all changes below:

| **Change** | **Location** | **Reviewer** |
| --- | --- | --- |
| Estimates framed as associations | Abstract, Methods, Results, Discussion | R4 Major #1 |
| Causal interpretation “suggestive” | Discussion Lim. 4, Conclusion | R4 Major #1 |
| Named missing confounders | Discussion Limitation 4 | R4 Major #1 |
| Cross-estimator consistency | Methods, Results, Supp. Table S2 | R4 Major #2 |
| SMD balance diagnostics | Methods, Results, Supp. Table S3 | R4 Major #3, Minor #4 |
| Weight diagnostics / ESS | Methods, Results, Supp. Table S4 | R4 Major #3 |
| Removed power analysis | Methods (removed), Discussion Lim. 5 | R4 Major #4 |
| Expanded weighting procedure | Methods | R4 Minor #1 |
| “Displaced group” terminology | Methods (throughout) | R4 Minor #2 |
| Temporal sequence clarification | Discussion Limitation 1 | R4 Minor #3, R2 Comment 1 |
| ATE vs. ATT interpretation | Discussion (new paragraph) | R2 Comment 5 |
| Integrated response rate arguments | Discussion (strengths paragraph) | R2 Comment 3 |
| Time-varying confounding | Discussion Limitation 1 | R2 Comment 1 |
| Dose-response dimensions | Discussion Limitation 3 | R2 Comment 2 |
| Table 3b label corrected | Results | R2 Comment 4 |
| Bootstrap CI description corrected | Methods, Abstract, Table notes | Editorial correction |
| MI description clarified | Methods | R2 Comment 6 |
| Abbreviations in tables | Tables 1, 2, 3a, 3b, S2–S4 | R3 |
