# Supplementary material for: Understanding the impact of natural disasters on post-traumatic stress disorder and depression symptoms: An examination of counterfactual displacement scenarios
Source: PLOS Ment Health. 2026 Jun 18;3(6):e0000317. doi: 10.1371/journal.pmen.0000317 (PMC13278393; doi:10.1371/journal.pmen.0000317)
Supplement: Peer review history

# Peer review: Decision Letter 0

**DOI:** 10.1371/journal.pmen.0000317.r001

**Authors:** Zahra Al-Khateeb

4 May 2025

PMEN-D-25-00135

Understanding the impact of natural disasters on post-traumatic stress disorder and depression symptoms: An examination of counterfactual displacement scenarios.

PLOS Mental Health

Dear Dr. Abba-Aji,

Thank you for submitting your manuscript to PLOS Mental Health. After careful consideration, we feel that it has merit but does not fully meet PLOS Mental Health’s publication criteria as it currently stands. Therefore, we invite you to submit a revised version of the manuscript that addresses the points raised during the review process.

==============================

The manuscript has been evaluated by one reviewers, and their comments are available below. The reviewers have raised a number of concerns that need attention. Specifically, They requested additional details related to the demographics of participant and the steps taken to mitigate selection bias. They also asked how the results presented in this work will advance/refine existing theories of disaster‑related displacement and trauma.

Could you please carefully revise the manuscript to address all comments raised?

Please note that we have only been able to secure a single reviewer to assess your manuscript. We are issuing a decision on your manuscript at this point to prevent further delays in the evaluation of your manuscript. Please be aware that the editor who handles your revised manuscript might find it necessary to invite additional reviewers to assess this work once the revised manuscript is submitted. However, we will aim to proceed on the basis of this single review if possible.

==============================

Please submit your revised manuscript by Jun 16 2025 11:59PM. If you will need more time than this to complete your revisions, please reply to this message or contact the journal office at mentalhealth@plos.org. When you’re ready to submit your revision, log on to https://www.editorialmanager.com/pmen/ and select the 'Submissions Needing Revision' folder to locate your manuscript file.

Please include the following items when submitting your revised manuscript:

- A rebuttal letter that responds to each point raised by the editor and reviewer(s). You should upload this letter as a separate file labeled 'Response to Reviewers'.
- A marked-up copy of your manuscript that highlights changes made to the original version. You should upload this as a separate file labeled 'Revised Manuscript with Track Changes'.
- An unmarked version of your revised paper without tracked changes. You should upload this as a separate file labeled 'Manuscript'.

Guidelines for resubmitting your figure files are available below the reviewer comments at the end of this letter.

We look forward to receiving your revised manuscript.

Kind regards,

Zahra Al-Khateeb, Ph.D

Staff Editor

PLOS Mental Health

Journal Requirements:

1. Please provide additional details regarding participant consent. In the ethics statement in the Methods and online submission information, please ensure that you have specified (1) whether consent was informed and (2) what type you obtained (for instance, written or verbal, and if verbal, how it was documented and witnessed). If your study included minors, state whether you obtained consent from parents or guardians. If the need for consent was waived by the ethics committee, please include this information.

If you are reporting a retrospective study of medical records or archived samples, please ensure that you have discussed whether all data were fully anonymized before you accessed them and/or whether the IRB or ethics committee waived the requirement for informed consent. If patients provided informed written consent to have data from their medical records used in research, please include this information.

2. Please insert an Ethics Statement at the beginning of your Methods section, under a subheading 'Ethics Statement'. It must include:

1) The name(s) of the Institutional Review Board(s) or Ethics Committee(s)

2) The approval number(s), or a statement that approval was granted by the named board(s)

3) (for human participants/donors) - A statement that formal consent was obtained (must state whether verbal/written) OR the reason consent was not obtained (e.g. anonymity). NOTE: If child participants, the statement must declare that formal consent was obtained from the parent/guardian.

3. Please provide separate figure files in .tif or .eps format.

For more information about figure files please see our guidelines:

https://journals.plos.org/mentalhealth/s/figures

https://journals.plos.org/mentalhealth/s/figures#loc-file-requirements

4. In the online submission form, you indicated that “Data is available upon request.”.

All PLOS journals now require all data underlying the findings described in their manuscript to be freely available to other researchers, either

1. In a public repository,

2. Within the manuscript itself, or

3. Uploaded as supplementary information.

This policy applies to all data except where public deposition would breach compliance with the protocol approved by your research ethics board. If your data cannot be made publicly available for ethical or legal reasons (e.g., public availability would compromise patient privacy), please explain your reasons by return email and your exemption request will be escalated to the editor for approval. Your exemption request will be handled independently and will not hold up the peer review process, but will need to be resolved should your manuscript be accepted for publication. One of the Editorial team will then be in touch if there are any issues.

Additional Editor Comments (if provided):

[Note: HTML markup is below. Please do not edit.]

Reviewers' comments:

Reviewer's Responses to Questions

**Comments to the Author**

1. Does this manuscript meet PLOS Mental Health’s publication criteria? Is the manuscript technically sound, and do the data support the conclusions? The manuscript must describe methodologically and ethically rigorous research with conclusions that are appropriately drawn based on the data presented.

Reviewer #1: Partly

**********

2. Has the statistical analysis been performed appropriately and rigorously?

Reviewer #1: Yes

**********

3. Have the authors made all data underlying the findings in their manuscript fully available (please refer to the Data Availability Statement at the start of the manuscript PDF file)?

The PLOS Data policy requires authors to make all data underlying the findings described in their manuscript fully available without restriction, with rare exception. The data should be provided as part of the manuscript or its supporting information, or deposited to a public repository. For example, in addition to summary statistics, the data points behind means, medians and variance measures should be available. If there are restrictions on publicly sharing data—e.g. participant privacy or use of data from a third party—those must be specified.

Reviewer #1: Yes

**********

4. Is the manuscript presented in an intelligible fashion and written in standard English?

PLOS Mental Health does not copyedit accepted manuscripts, so the language in submitted articles must be clear, correct, and unambiguous. Any typographical or grammatical errors should be corrected at revision, so please note any specific errors here.

Reviewer #1: Yes

**********

5. Review Comments to the Author

Please use the space provided to explain your answers to the questions above. You may also include additional comments for the author, including concerns about dual publication, research ethics, or publication ethics. (Please upload your review as an attachment if it exceeds 20,000 characters)

Reviewer #1: The authors examined the cross-sectional association between residential relocation and trauma-related psychopathology. While they offered valuable insight on the risk of PTSD/depression after displacement by considering the counterfactual scenario, the low response rate (approximately 10%) limits the confidence in the representativeness of the target population and raises concerns about selection bias.

1. Can you show a table comparing key demographic and clinical characteristics of the target population and your analytic sample? It might help readers understand what population you analyzed.

2. Weighting adjustment is necessary to mitigate the estimation bias because of the low response rate.

3. Please justify how your results advance or refine existing theories of disaster‑related displacement and trauma, despite the remaining methodological limitations (low response rate).

**********

6. PLOS authors have the option to publish the peer review history of their article (what does this mean?). If published, this will include your full peer review and any attached files.

**Do you want your identity to be public for this peer review?**   If you choose “no”, your identity will remain anonymous but your review may still be made public.

For information about this choice, including consent withdrawal, please see our Privacy Policy.

Reviewer #1: No

**********

[NOTE: If reviewer comments were submitted as an attachment file, they will be attached to this email and accessible via the submission site. Please log into your account, locate the manuscript record, and check for the action link "View Attachments". If this link does not appear, there are no attachment files.]

While revising your submission, please upload your figure files to the Preflight Analysis and Conversion Engine (PACE) digital diagnostic tool, https://pacev2.apexcovantage.com/. PACE helps ensure that figures meet PLOS requirements. To use PACE, you must first register as a user. Registration is free. Then, login and navigate to the UPLOAD tab, where you will find detailed instructions on how to use the tool. If you encounter any issues or have any questions when using PACE, please email PLOS at figures@plos.org. Please note that Supporting Information files do not need this step.

# Author comment: Author response to Decision Letter 1

**DOI:** 10.1371/journal.pmen.0000317.r002

28 Jun 2025

Attachment Submitted filename: Response to Reviewers_PLOS_MH.docx

# Peer review: Decision Letter 1

**DOI:** 10.1371/journal.pmen.0000317.r003

**Authors:** Zahra Al-Khateeb, Paolo Raile

17 Jul 2025

PMEN-D-25-00135R1

Understanding the impact of natural disasters on post-traumatic stress disorder and depression symptoms: An examination of counterfactual displacement scenarios.

PLOS Mental Health

Dear Dr. Abba-Aji,

Thank you for submitting your manuscript to PLOS Mental Health. After careful consideration, we have decided that your manuscript does not meet our criteria for publication and must therefore be rejected.

Specifically:

After the revision, the main concerns regarding scientific validity remains. You can of course revise the article thoroughly and submit it to another journal.

We are sorry that we cannot be more positive on this occasion. We very much appreciate your wish to present your work in one of PLOS's Open Access publications. Thank you for your support, and we hope that you will consider PLOS Mental Health for other submissions in the future.

Yours sincerely,

Paolo Raile

Academic Editor

PLOS Mental Health

Additional Editor Comments (if provided):

Thank you for your submission. Unfortunately, we have to reject the manuscript since even after a revision, the strong concerns regarding the scientific validity remains.

[Note: HTML markup is below. Please do not edit.]

Reviewers' comments:

Reviewer's Responses to Questions

**Comments to the Author**

1. If the authors have adequately addressed your comments raised in a previous round of review and you feel that this manuscript is now acceptable for publication, you may indicate that here to bypass the “Comments to the Author” section, enter your conflict of interest statement in the “Confidential to Editor” section, and submit your "Accept" recommendation.

Reviewer #1: (No Response)

**********

2. Does this manuscript meet PLOS Mental Health’s publication criteria? Is the manuscript technically sound, and do the data support the conclusions? The manuscript must describe methodologically and ethically rigorous research with conclusions that are appropriately drawn based on the data presented.

Reviewer #1: No

**********

3. Has the statistical analysis been performed appropriately and rigorously?

Reviewer #1: Yes

**********

4. Have the authors made all data underlying the findings in their manuscript fully available (please refer to the Data Availability Statement at the start of the manuscript PDF file)?

The PLOS Data policy requires authors to make all data underlying the findings described in their manuscript fully available without restriction, with rare exception. The data should be provided as part of the manuscript or its supporting information, or deposited to a public repository. For example, in addition to summary statistics, the data points behind means, medians and variance measures should be available. If there are restrictions on publicly sharing data—e.g. participant privacy or use of data from a third party—those must be specified.

Reviewer #1: No

**********

5. Is the manuscript presented in an intelligible fashion and written in standard English?

PLOS Mental Health does not copyedit accepted manuscripts, so the language in submitted articles must be clear, correct, and unambiguous. Any typographical or grammatical errors should be corrected at revision, so please note any specific errors here.

Reviewer #1: Yes

**********

6. Review Comments to the Author

Please use the space provided to explain your answers to the questions above. You may also include additional comments for the author, including concerns about dual publication, research ethics, or publication ethics. (Please upload your review as an attachment if it exceeds 20,000 characters)

Reviewer #1: The authors attempted to address the sample bias inherent in a 10% response rate; however, Table S1 presents only three variables—gender, age, and race—and fails to provide representative data for other key socioeconomic factors such as income, educational attainment, and residential area. As a result, potential sources of bias remain unaddressed, and a more equitable presentation of information is required.

Furthermore, even after weighting, racial imbalances persist. Under these conditions, systematic bias will continue to affect the analyses as a whole, undermining the validity of the conclusions.

Therefore, at this stage, the sample’s representativeness of the target population cannot be deemed adequate, and there is a high risk that the published estimates will be biased. I decided that the manuscript’s conclusions cannot be considered reliable and that rejection is appropriate.

**********

7. PLOS authors have the option to publish the peer review history of their article (what does this mean?). If published, this will include your full peer review and any attached files.

**Do you want your identity to be public for this peer review?**   If you choose “no”, your identity will remain anonymous but your review may still be made public.

For information about this choice, including consent withdrawal, please see our Privacy Policy.

Reviewer #1: No

**********

[NOTE: If reviewer comments were submitted as an attachment file, they will be attached to this email and accessible via the submission site. Please log into your account, locate the manuscript record, and check for the action link "View Attachments". If this link does not appear, there are no attachment files.]

# Author comment: Author response to Decision Letter 2

**DOI:** 10.1371/journal.pmen.0000317.r004

20 Oct 2025

Attachment Submitted filename: Response to Editor_Reviewer _PLOS_MH_2.docx

# Peer review: Decision Letter 2

**DOI:** 10.1371/journal.pmen.0000317.r005

**Authors:** Zahra Al-Khateeb, Paolo Raile, Paolo Raile

15 Dec 2025

PMEN-D-25-00135R2

Understanding the impact of natural disasters on post-traumatic stress disorder and depression symptoms: An examination of counterfactual displacement scenarios.

PLOS Mental Health

Dear Dr. Abba-Aji,

Thank you for submitting your manuscript to PLOS Mental Health. After careful consideration, we feel that it has merit but does not fully meet PLOS Mental Health’s publication criteria as it currently stands. Therefore, we invite you to submit a revised version of the manuscript that addresses the points raised during the review process.

==============================

EDITOR: Thank you for submitting your manuscript to PLIS Mental Health. Please read the reviewer’s comments carefully and revise your manuscript.

==============================

Please submit your revised manuscript by Jan 29 2026 11:59PM. If you will need more time than this to complete your revisions, please reply to this message or contact the journal office at mentalhealth@plos.org. When you're ready to submit your revision, log on to https://www.editorialmanager.com/pmen/ and select the 'Submissions Needing Revision' folder to locate your manuscript file.

Please include the following items when submitting your revised manuscript:

- A letter that responds to each point raised by the editor and reviewer(s). You should upload this letter as a separate file labeled 'Response to Reviewers'.
- A marked-up copy of your manuscript that highlights changes made to the original version. You should upload this as a separate file labeled 'Revised Manuscript with Track Changes'.
- An unmarked version of your revised paper without tracked changes. You should upload this as a separate file labeled 'Manuscript'.

Guidelines for resubmitting your figure files are available below the reviewer comments at the end of this letter.

We look forward to receiving your revised manuscript.

Kind regards,

Paolo Raile

Academic Editor

PLOS Mental Health

Journal Requirements:

1. If the reviewer comments include a recommendation to cite specific previously published works, please review and evaluate these publications to determine whether they are relevant and should be cited. There is no requirement to cite these works unless the editor has indicated otherwise.

Additional Editor Comments (if provided):

Thank you for submitting your manuscript to PLIS Mental Health. Please read the reviewer’s comments carefully and revise your manuscript.

[Note: HTML markup is below. Please do not edit.]

Reviewers' comments:

Reviewer's Responses to Questions

**Comments to the Author**

1. If the authors have adequately addressed your comments raised in a previous round of review and you feel that this manuscript is now acceptable for publication, you may indicate that here to bypass the “Comments to the Author” section, enter your conflict of interest statement in the “Confidential to Editor” section, and submit your "Accept" recommendation.

Reviewer #2: All comments have been addressed

Reviewer #3: All comments have been addressed

Reviewer #4: (No Response)

**********

2. Does this manuscript meet PLOS Mental Health’s publication criteria? Is the manuscript technically sound, and do the data support the conclusions? The manuscript must describe methodologically and ethically rigorous research with conclusions that are appropriately drawn based on the data presented.

Reviewer #2: Yes

Reviewer #3: Yes

Reviewer #4: Partly

**********

3. Has the statistical analysis been performed appropriately and rigorously?

Reviewer #2: Yes

Reviewer #3: Yes

Reviewer #4: No

**********

4. Have the authors made all data underlying the findings in their manuscript fully available (please refer to the Data Availability Statement at the start of the manuscript PDF file)?

The PLOS Data policy requires authors to make all data underlying the findings described in their manuscript fully available without restriction, with rare exception. The data should be provided as part of the manuscript or its supporting information, or deposited to a public repository. For example, in addition to summary statistics, the data points behind means, medians and variance measures should be available. If there are restrictions on publicly sharing data—e.g. participant privacy or use of data from a third party—those must be specified.

Reviewer #2: Yes

Reviewer #3: Yes

Reviewer #4: No

**********

5. Is the manuscript presented in an intelligible fashion and written in standard English?

PLOS Mental Health does not copyedit accepted manuscripts, so the language in submitted articles must be clear, correct, and unambiguous. Any typographical or grammatical errors should be corrected at revision, so please note any specific errors here.

Reviewer #2: Yes

Reviewer #3: Yes

Reviewer #4: Yes

**********

6. Review Comments to the Author

Please use the space provided to explain your answers to the questions above. You may also include additional comments for the author, including concerns about dual publication, research ethics, or publication ethics. (Please upload your review as an attachment if it exceeds 20,000 characters)

Reviewer #2: This paper addresses a critically important and timely public health issue: the causal effect of forced displacement following a natural disaster on mental health (PTSD and depression). The author team employs an advanced causal inference method (AIPW and IPWRA within a counterfactual framework) to analyze cross-sectional survey data from Houston residents affected by Hurricane Harvey. This represents a significant methodological advancement, surpassing many studies in this field that rely on descriptive statistics or traditional regression analyses. While the study has numerous strengths, there is room for improvement in the presentation of the research design and analytical details to further enhance its persuasiveness and clarity.

II. Specific Comments on Research Design

Primary Limitation: Cross-sectional Design and Time Lag

Comment: The authors correctly acknowledge the limitations of the cross-sectional design in the discussion. However, this issue warrants deeper consideration. Hurricane Harvey occurred in 2017, while the mental health data were collected in 2020-2021, creating a 3-4 year gap. Although the authors have diligently ensured that covariates reflect circumstances at or before the disaster, the potential influence of other life events during this period (e.g., job loss, family changes, new traumatic experiences) cannot be ruled out. These events might have concurrently affected mental health status and been associated with prolonged displacement status. This represents a potential source of unmeasured time-varying confounding. It is recommended that the authors more explicitly highlight this point in the limitations section of the discussion.

Definition and Measurement of Exposure

Comment: Operationalizing "forced displacement" as a binary variable (yes/no) is clear but simplifies a complex reality. While the authors acknowledge this in the discussion, further elaboration would be beneficial. Dimensions such as the duration of displacement, the number of relocations, the stability of temporary housing, and separation from family could potentially constitute a dose-response relationship. Although the current data may not support such granular analysis, emphasizing the importance of these dimensions in the discussion and suggesting them for future research would make the paper more forward-looking.

Sample Representativeness and Response Rate

The authors have provided a very thorough and professional defense regarding this issue in their response to the editor. Comparing the 11.4% response rate to national surveys like NSDUH is reasonable; emphasizing that internal validity (achieved through covariate balance) is more central to causal inference than external validity (sample representativeness) is entirely correct. However, I would recommend that the core arguments from the response letter—specifically the comparison with NSDUH, the primacy of internal validity, and the efforts to address concerns via weighting and sensitivity analyses—be concisely integrated into the Methods section or the limitations paragraph of the manuscript's Discussion. This would proactively address potential reader concerns and make the manuscript more self-contained and complete.

Table Issue

In the results section, the titles for Table 3a and Table 3b are duplicated. The first table (ATE) is titled "Table 3a. Population-level effects...", while the second table (ATT) is also titled "Table 3a. Individual-level effects...". This appears to be a typesetting error.

Interpretation of ATE and ATT Results

The authors report both the Average Treatment Effect (ATE) and the Average Treatment Effect on the Treated (ATT) and provide clear definitions. I would suggest that the discussion could briefly compare and interpret the difference between the ATE and ATT estimates. For instance, for PTSD, the ATE (5.88) is slightly larger than the ATT (5.43), whereas for depression, the ATE (1.93) is slightly smaller than the ATT (2.12). What might these slight differences suggest? Could it indicate that individuals who ended up being displaced possess certain unobserved characteristics that make their response to displacement slightly different from that of the general population? This is not to demand a definitive conclusion but offering a brief reflection on this discrepancy would demonstrate a deeper engagement with the results.

Handling of Missing Data

Comment: The use of Multiple Imputation by Chained Equations (MICE) for handling <5% missing data is standard and appropriate. For the sake of absolute clarity, it is recommended that the Methods section explicitly state that all primary analyses (AIPW/IPWRA) were run on each of the 15 imputed datasets separately, with the results then pooled (e.g., using Rubin's rules).

Reviewer #3: The authors have adequately addressed all of the comments raised by the previous reviewer. The remaining issue to be resolved is that all abbreviations (e.g., IQR, CI, etc.) should be spelled out in full not only in the main text but also in all tables.

Reviewer #4: I appreciate the opportunity to review this intellectually engaging and timely manuscript. This manuscript provides a meaningful exploration of how post-disaster displacement influences mental health outcomes such as PTSD and depression symptoms. However, several major methodological and interpretive issues require substantial revision before the study’s conclusions can be considered robust.

# major

1. line 355

The set of covariates used to adjust for confounding (gender, age, education, race/ethnicity, marital status, living condition, and self-reported physical health) is rather limited for causal inference in the context of disaster-related mental health. Critically, key potential confounders such as prior trauma history, pre-disaster mental health status, detailed socioeconomic indicators (e.g., employment, housing insecurity), social support, and severity of disaster exposure are not included. Many of these factors are likely to influence both the likelihood of displacement and subsequent PTSD and depression symptoms. As a result, the assumption of no unmeasured confounding is unlikely to hold, and the estimated ‘effects’ of displacement should be interpreted as associations rather than strong causal effects.

The authors argue that unmeasured confounding is unlikely based on konfound sensitivity analyses and the use of doubly robust estimators. However, this conclusion appears overstated. The konfound framework provides a highly simplified assessment and does not account for realistic confounders relevant to disaster-related mental health, such as prior trauma exposure, baseline symptoms, socioeconomic instability, or pre-disaster mental health treatment. Several of these unmeasured variables are plausibly correlated with both displacement and mental health outcomes at levels exceeding the reported 0.26 threshold, which challenges the claim that such confounding is ‘implausible.’ Moreover, double robustness addresses model misspecification but does not protect against omitted variable bias. Overall, the assertion that the results are robust to unmeasured confounding is insufficiently supported and should be substantially qualified.

Accordingly, this issue should be treated as a key limitation of the study, and the authors are encouraged to substantially tone down their claims in this section.

2-1. line 183

The description of AIPW and IPWRA is incomplete and partially inaccurate. The authors incorrectly imply that IPWRA estimates the ATT specifically, although both AIPW and IPWRA can target either the ATE or ATT depending on the estimand defined. Moreover, the causal estimand being estimated (ATE vs ATT) is not explicitly stated, which is essential for interpretation. The methodological distinction between AIPW and IPWRA is not clearly articulated, and the justification for choosing these methods is insufficient.

2-2. line 272

Although both AIPW and IPWRA can estimate either ATE or ATT depending on the specification, the manuscript assigns ATE estimation exclusively to AIPW and ATT estimation exclusively to IPWRA without providing methodological justification. Because neither estimator is inherently tied to a specific estimand, this pairing appears arbitrary. The authors should clarify why different estimators were used for different estimands, or alternatively, provide results showing that both AIPW and IPWRA yield consistent estimates of both ATE and ATT.

3. line 274

The balance and robustness section provides limited evidence of adequate covariate balance. Reliance solely on an overidentification test and PS distribution plots is insufficient, as these do not substitute for standardized mean differences (SMDs), effective sample size after weighting, or examination of extreme weights. The extremely high p-values (>0.95) from the χ² tests should be interpreted cautiously, as they may reflect variance reduction rather than true substantive balance. Additionally, konfound results do not address key omitted confounders relevant to disaster-related mental health (e.g., prior trauma history, baseline mental health, socioeconomic stressors), limiting the validity of the robustness claims. Overall, the balance diagnostics and sensitivity analyses are not comprehensive enough to fully support the causal interpretations presented.

4. line 137

The current sample size and power subsection relies on a post hoc power calculation, which offers limited inferential value and may be misleading. Post hoc power is largely determined by the observed effect and final sample size, and it does not account for the reduction in effective sample size introduced by the propensity score procedures. Rather than including this calculation, it would be more appropriate to remove this subsection and acknowledge the inability to conduct an a priori sample size estimation—possibly with the limitation of the low response rate—as a study limitation

# minor

1. line 98

The description of the weighting procedure lacks essential methodological details required for reproducibility and assessment of potential bias. Although the authors state that complex survey weights and post-stratification were applied, the specific steps, models, calibration methods, and diagnostics are insufficiently reported. This limits transparency and undermines the strength of their representativeness claim.

2. line 112

Referring to displaced individuals as a ‘treatment group’ is conceptually inappropriate, as displacement is not an intervention but an externally imposed exposure. This terminology may mislead readers by implying an experimental design. I recommend renaming this group as the ‘exposed group’ or ‘displaced group’ to maintain clarity and conceptual accuracy.

3. line 34

The authors state that displacement-related variables and covariates represent conditions prior to or at the time of displacement. However, because exposure and covariates were collected concurrently in 2020, the manuscript does not clearly explain how pre-displacement status was established. Without explicit methodological detail demonstrating that each covariate reflects pre-2017 conditions, this claim remains unverified. The authors should clarify how recall bias, post-displacement changes, or time-varying confounders were addressed. Otherwise, the statement that temporal sequence was ‘carefully ensured’ may overstate the strength of the evidence.

4. line 186

The overidentification test alone does not provide sufficient evidence of covariate balance.

Balance evaluation typically relies on standardized mean differences (SMDs), effective sample size (ESS), and examination of weight distributions. Without these diagnostics, the claim of ‘adequate balance’ is not fully supported.

**********

7. PLOS authors have the option to publish the peer review history of their article (what does this mean?). If published, this will include your full peer review and any attached files.

**Do you want your identity to be public for this peer review?**   If you choose “no”, your identity will remain anonymous but your review may still be made public.

For information about this choice, including consent withdrawal, please see our Privacy Policy.

Reviewer #2: No

Reviewer #3: No

Reviewer #4: No

**********

[NOTE: If reviewer comments were submitted as an attachment file, they will be attached to this email and accessible via the submission site. Please log into your account, locate the manuscript record, and check for the action link "View Attachments". If this link does not appear, there are no attachment files.]

-->Figure Resubmissions:

While revising your submission, we strongly recommend that you use PLOS’s NAAS tool (https://ngplosjournals.pagemajik.ai/artanalysis) to test your figure files. NAAS can convert your figure files to the TIFF file type and meet basic requirements (such as print size, resolution), or provide you with a report on issues that do not meet our requirements and that NAAS cannot fix.

After uploading your figures to PLOS’s NAAS tool - https://ngplosjournals.pagemajik.ai/artanalysis, NAAS will process the files provided and display the results in the "Uploaded Files" section of the page as the processing is complete. If the uploaded figures meet our requirements (or NAAS is able to fix the files to meet our requirements), the figure will be marked as "fixed" above. If NAAS is unable to fix the files, a red "failed" label will appear above. When NAAS has confirmed that the figure files meet our requirements, please download the file via the download option, and include these NAAS processed figure files when submitting your revised manuscript.

# Author comment: Author response to Decision Letter 3

**DOI:** 10.1371/journal.pmen.0000317.r006

18 Apr 2026

Attachment Submitted filename: PMEN_R2_Response_to_Reviewers_FINAL.docx

# Peer review: Decision Letter 3

**DOI:** 10.1371/journal.pmen.0000317.r007

**Authors:** Zahra Al-Khateeb, Paolo Raile, Paolo Raile, Paolo Raile

1 Jun 2026

Understanding the impact of natural disasters on post-traumatic stress disorder and depression symptoms: An examination of counterfactual displacement scenarios.

PMEN-D-25-00135R3

Dear Dr. Abba-Aji,

We are pleased to inform you that your manuscript 'Understanding the impact of natural disasters on post-traumatic stress disorder and depression symptoms: An examination of counterfactual displacement scenarios.' has been provisionally accepted for publication in PLOS Mental Health.

Before your manuscript can be formally accepted you will need to complete some formatting changes, which you will receive in a follow up email. A member of our team will be in touch with a set of requests.

Please note that your manuscript will not be scheduled for publication until you have made the required changes, so a swift response is appreciated.

IMPORTANT: The editorial review process is now complete. PLOS will only permit corrections to spelling, formatting or significant scientific errors from this point onwards. Requests for major changes, or any which affect the scientific understanding of your work, will cause delays to the publication date of your manuscript.

If your institution or institutions have a press office, please notify them about your upcoming paper to help maximize its impact. If they'll be preparing press materials, please inform our press team as soon as possible -- no later than 48 hours after receiving the formal acceptance. Your manuscript will remain under strict press embargo until 2 pm Eastern Time on the date of publication. For more information, please contact mentalhealth@plos.org.

Thank you again for supporting Open Access publishing; we are looking forward to publishing your work in PLOS Mental Health.

Best regards,

Paolo Raile

Academic Editor

PLOS Mental Health

***********************************************************

Thank you for submitting the revised manuscript. I am happy to inform you that the manuscript can now be accepted as it is.

Reviewer Comments (if any, and for reference):

Reviewer's Responses to Questions

**Comments to the Author**

1. If the authors have adequately addressed your comments raised in a previous round of review and you feel that this manuscript is now acceptable for publication, you may indicate that here to bypass the “Comments to the Author” section, enter your conflict of interest statement in the “Confidential to Editor” section, and submit your "Accept" recommendation.

Reviewer #2: All comments have been addressed

Reviewer #4: All comments have been addressed

**********

2. Does this manuscript meet PLOS Mental Health’s publication criteria? Is the manuscript technically sound, and do the data support the conclusions? The manuscript must describe methodologically and ethically rigorous research with conclusions that are appropriately drawn based on the data presented.

Reviewer #2: Yes

Reviewer #4: Yes

**********

3. Has the statistical analysis been performed appropriately and rigorously?

Reviewer #2: Yes

Reviewer #4: Yes

**********

4. Have the authors made all data underlying the findings in their manuscript fully available (please refer to the Data Availability Statement at the start of the manuscript PDF file)?

The PLOS Data policy requires authors to make all data underlying the findings described in their manuscript fully available without restriction, with rare exception. The data should be provided as part of the manuscript or its supporting information, or deposited to a public repository. For example, in addition to summary statistics, the data points behind means, medians and variance measures should be available. If there are restrictions on publicly sharing data—e.g. participant privacy or use of data from a third party—those must be specified.

Reviewer #2: Yes

Reviewer #4: No

**********

5. Is the manuscript presented in an intelligible fashion and written in standard English?

PLOS Mental Health does not copyedit accepted manuscripts, so the language in submitted articles must be clear, correct, and unambiguous. Any typographical or grammatical errors should be corrected at revision, so please note any specific errors here.

Reviewer #2: Yes

Reviewer #4: Yes

**********

6. Review Comments to the Author

Please use the space provided to explain your answers to the questions above. You may also include additional comments for the author, including concerns about dual publication, research ethics, or publication ethics. (Please upload your review as an attachment if it exceeds 20,000 characters)

Reviewer #2: None

Reviewer #4: The authors have adequately addressed the reviewer’s concerns and revised the manuscript accordingly. Thank you for your careful and thoughtful revisions.

**********

7. PLOS authors have the option to publish the peer review history of their article (what does this mean?). If published, this will include your full peer review and any attached files.

**Do you want your identity to be public for this peer review?**   If you choose “no”, your identity will remain anonymous but your review may still be made public.

For information about this choice, including consent withdrawal, please see our Privacy Policy.

Reviewer #2: No

Reviewer #4: No

**********
